# EDEM1 regulates the insulin mRNA level by inhibiting the endoplasmic reticulum stress-induced IRE1/JNK/c-Jun pathway

**DOI:** 10.1016/j.isci.2023.107956

**Published:** 2023-09-16

**Authors:** Petruta R. (Flintoaca) Alexandru, Gabriela N. Chiritoiu, Daniela Lixandru, Sabina Zurac, Constantin Ionescu-Targoviste, Stefana M. Petrescu

**Affiliations:** 1 Department of Molecular Cell Biology, Institute of Biochemistry, Romanian Academy, 060031 Bucharest, Romania; 2 Department of Biochemistry, “Carol Davila” University of Medicine and Pharmacy, 050474 Bucharest, Romania; 3 Department of Physiology, “Carol Davila” University of Medicine and Pharmacy, 050474 Bucharest, Romania; 4 N. C. Paulescu Institute, Bucharest, Romania

**Keywords:** Biochemistry, Physiology, Molecular biology, Cell biology

## Abstract

Pancreatic beta cells produce and secrete insulin as a response to rises in blood glucose. Despite the advances in understanding glucose-regulated insulin transcription and translation the mechanisms triggering the synthesis of new insulin molecules are still incompletely described. In this report, we identify EDEM1 as a new modulator of insulin synthesis and secretion. In the presence of EDEM1, INS-1E cells secrete significantly more insulin upon glucose stimulation compared to control cells. We found that overexpression of EDEM1 inhibited the IRE1/JNK/c-Jun pathway, leading to an increase in the insulin mRNA level. Similarly, EDEM1 transduced human islets secreted significantly more insulin upon stimulation. Furthermore, EDEM1 improved insulin secretion restoring normoglycemia and glucose tolerance in diabetic rats. We propose EDEM1 as a regulator of the UPR via IRE1/XBP1s and IRE1/JNK/c-Jun signaling cascades and insulin transcription in pancreatic β-cells, supporting EDEM1 as a potential target for the treatment of diabetes.

## Highlights

• EDEM1 is a new modulator of insulin synthesis and secretion in human islets
• EDEM1 promotes insulin gene transcription
• Increased glucose tolerance in the presence of EDEM1 in diabetic rats
• EDEM1 regulates the UPR via IRE1/XBP1s and IRE1/JNK/c-Jun signaling cascades

## Graphical abstract

**Figure:**
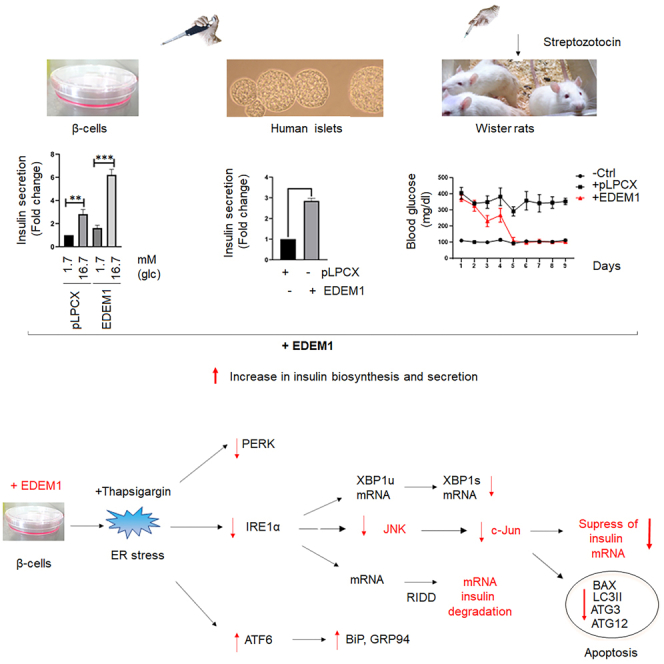

## Introduction

Insulin is a peptide hormone that controls glucose homeostasis. The hormone is secreted by pancreatic beta cells as a response to rises in blood glucose and regulates the glucose level by supporting its cellular uptake.^1^ Synthesized as preproinsulin, the polypeptide is converted to proinsulin.^2^^,^^3^ Within the endoplasmic reticulum (ER) lumen, proinsulin undergoes folding into a native conformation which is stabilized upon the formation of three disulphide bonds.^4^^,^^5^ Properly folded proinsulin is transported through the secretory pathway and sorted into immature clathrin-coated secretory vesicles that undergo a maturation process.^6^^,^^7^^,^^8^ Proteolytic maturation of insulin accompanied by the removal and secretion of the C-peptide corresponds to the maturation of secretory vesicles during the acidification step.^9^ In these secretory vesicles insulin which is packed and stored and released into the bloodstream upon glucose stimulation.^10^ Protein quality control of the ER is crucial in maintaining cell protein homeostasis and the efficiency of the secretory pathway in pancreatic β−cells.^11^ The accumulation of misfolded proinsulin molecules initiates the UPR (unfolded protein response) that in conjunction with the endoplasmic reticulum-associated degradation pathway (ERAD) regulates the ER homeostasis.^12^

The UPR is a complex signal transduction pathway initiated by the activation of three ER-localized transmembrane sensors: inositol-requiring enzyme 1α (IRE1α), protein kinase RNA-activated (PKR)-like kinase (PERK) and activating transcription factor 6 (ATF6).^13^ In the absence of stress they are maintained in an inactive state by the binding of the chaperone BiP to their luminal domains. However, the accumulation of misfolded polypeptides favors BiP binding to these polypeptides, thus releasing the UPR sensors that induce distinct signaling cascades activating downstream effectors to restore endoplasmic and mitochondrial homeostasis.^14^^,^^15^ Chronic exposure of β-cells to high glucose causes ER stress and hyperactivation of IRE1, and suppression of insulin gene expression. Activated IRE1 then splices X-box binding protein-1 (XBP1) mRNA and upregulation of UPR genes. Phosphorylated IRE1 induces a cascade of events that activate the MAP3K, ASK1, and ultimately JNK (c-Jun N-terminal kinase).^16^ Several studies have reported an increase in JNK activity in response to diabetes and accumulation of the misfolded proteins in the ER.^17^^,^^18^^,^^19^^,^^20^ By activating c-Jun, phosphorylated JNK is involved in the control of insulin transcription,^21^ as it was shown that the activation of JNK pathway decreased PDX1 DNA binding activity and suppression of the insulin gene transcription.^22^^,^^23^ This complex cascade of UPR signaling activates a number of ER resident proteins during the crosstalks with the protein quality control and the ERAD (endoplasmic reticulum-associated degradation) pathway. A component of this pathway is the EDEM family, gene including EDEM1 (ER degradation-enchancing alpha-mannosidase-like protein1), EDEM2 and EDEM3 that is upregulated during UPR and able to recognize and deliver misfolded proteins to degradation by the ERAD pathway.^24^^,^^25^^,^^26^^,^^29^^,^^27^ Here, we have investigated the role of EDEM proteins in the insulin synthesis and secretion. We showed that overexpression of EDEM1 in INS-1E β−cells led to increased insulin content and secretion and conversely, EDEM1 silencing led to decreased insulin expression. Insulin secretion was significantly improved in human pancreatic islets overexpressing EDEM1, while diabetic mice improved their glucose tolerance upon treatment with EDEM1. Mechanistically, we found that overexpression of EDEM1 inhibited the IRE1/XBP1 signaling pathway preventing the degradation of insulin mRNA by IRE1 and at the same time reduced the expression of P-*c*-Jun, a suppressor of the insulin gene expression, resulting in an increase in insulin mRNA in pancreatic beta cells.

## Results

### Regulation of insulin cell content and insulin secretion by EDEM1

**Figure 1:**
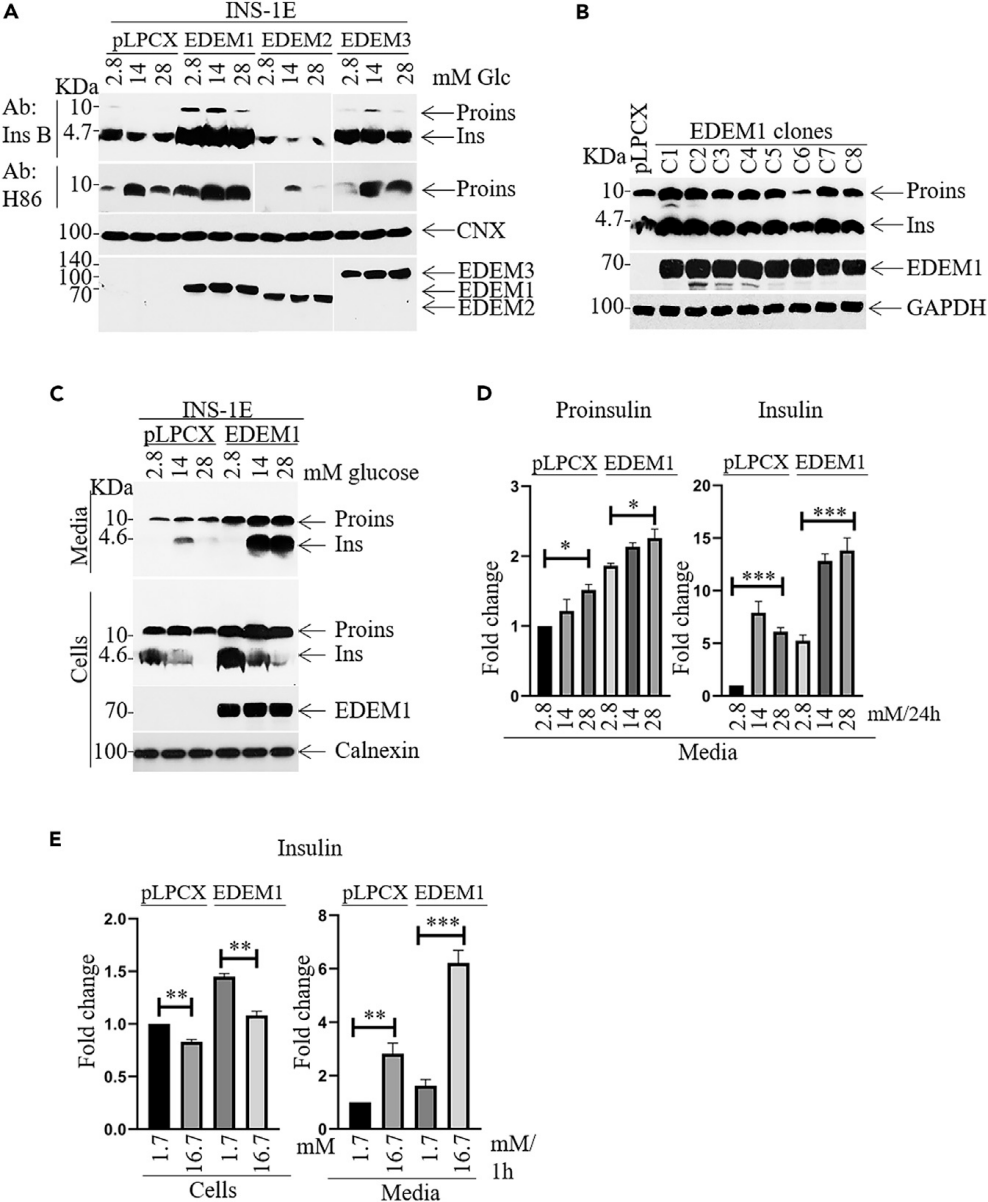
EDEM1 overexpression increases insulin content and secretion in insulinoma cells (A) INS-1E-pLPCX cells and cells stably expressing EDEM1, EDEM2 and EDEM3 were seeded 48h before starvation in medium without glucose for 1h and exposed to a range of glucose concentrations (2.8mM, 14mM and 28mM) for 24 h. Cells were analyzed by Tris/Tricine SDS-PAGE followed by immunoblotting with anti-proinsulin (H86), anti-insulin B (Ins B) antibodies and by Tris/Glycine SDS-PAGE followed by immunoblotting with specific antibodies for EDEMs. (B) The insulin level in eight cell lines derived from parental INS-1E cells, expressing different amount of EDEM1 protein were analyzed by Western Blotting with EDEM1 and insulin B antibodies. Cells were grown in RPMI-1640 medium with 11.2 mM glucose for 72h. (C) As in (A) but in addition, glucose-stimulated insulin secretion was analyzed in the presence of overexpressed EDEM1 or control cells, calnexin was used as loading control. (D) As in (C) but proinsulin and insulin secretion level were quantified by ELISA assay. (E) As in (D) but cells were seeded 72h before stimulation with glucose. After starvation in medium without glucose for 1h, cells were incubated with 1.7mM and 16.7 mM glucose for 1h. Cell lysates and secretion media were analyzed by ELISA assay. Data are represented as means ± SEM. Data are representative for at least three independent experiments.

To investigate the role of EDEM proteins in pancreatic β-cells we created INS-1E cell lines overexpressing EDEM1, EDEM2 and EDEM3, respectively.^16^^,^^29^ We analyzed the variations in the proinsulin and insulin intracellular level exposing cell clones expressing EDEM at basal (2.8mM) and high glucose concentrations (14 mM and 28mM) that stimulate insulin secretion. The Western blot analysis revealed an increased proinsulin and insulin content in the presence of EDEM1 and EDEM3 rather than EDEM2 (Figure 1A). Quantification of the 14mM glucose exposure revealed that EDEM1 showed the most prominent effect (Figure S1A).

To further evaluate the effect of EDEM1 upon proinsulin biosynthesis, we characterized more cell clones expressing EDEM1. We found that 87% of the isolated clones presented an increased proinsulin expression with an efficient conversion of proinsulin to insulin (Figure 1B and quantification at 14 mM glucose concentration in Figure S1B), suggesting that EDEM1 may play an important role in the insulin secretion regulation.

We further examined whether the Western blot data could be validated using as an alternative method the ELISA assay. Testing the insulin secreted by the INS-1E-EDEM1 clones previously characterized in Figure 1B we found that most of these INS-1E-EDEM1 clones secrete significantly more insulin in the extracellular environment compared to control cells (Figure S1C). We detected also proinsulin in the media, although at a considerably lower level than insulin. Both Western blot and ELISA assays of the clone INS-1E-EDEM1 C1 (further analyzed throughout this article) revealed that glucose-stimulated insulin secretion was also increased by EDEM1 and this increase was coordinated with the increasing glucose concentrations (Figures 1C and 1D and quantification at 14mM glucose in Figure S1D).

**Figure 2:**
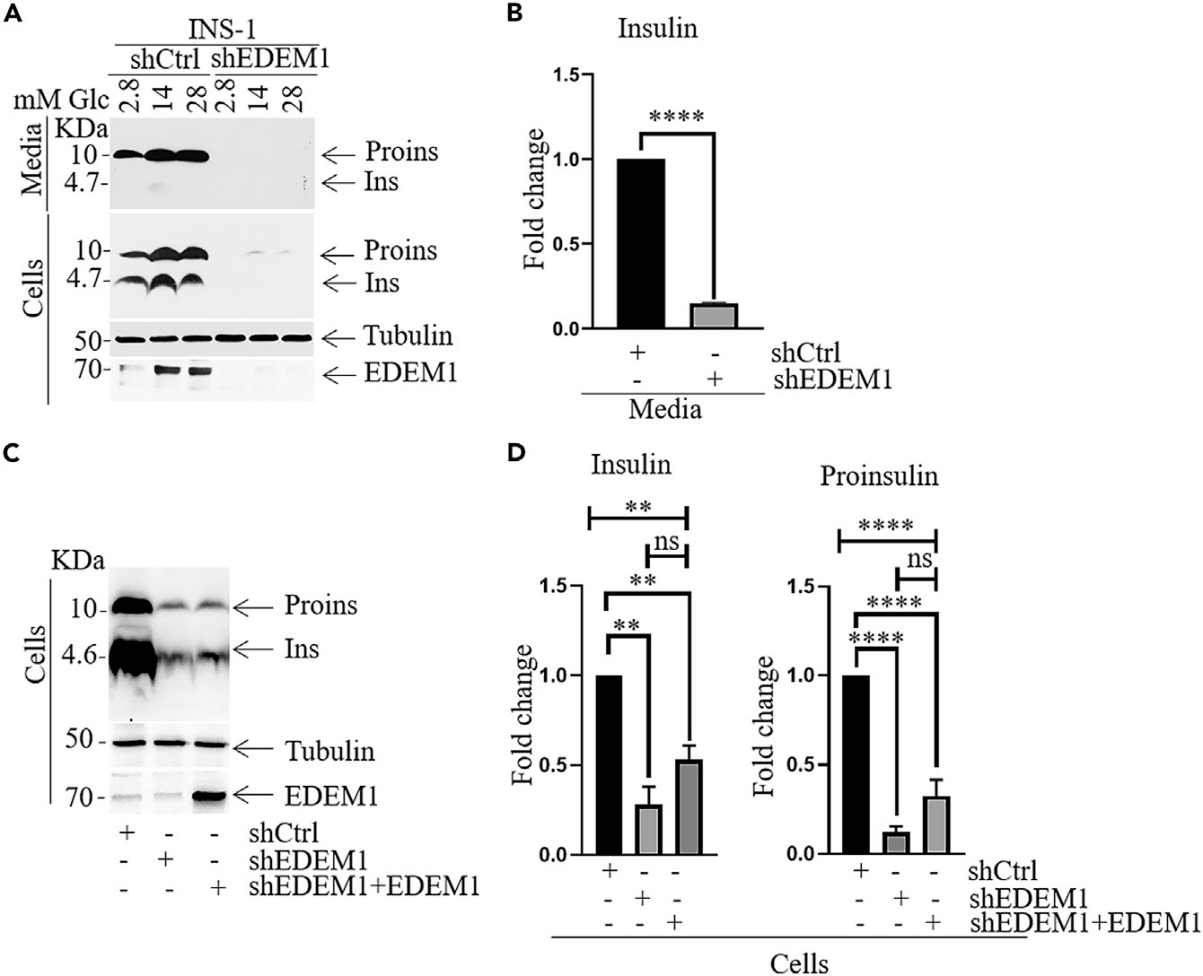
EDEM1 silencing decreases insulin content and secretion in INS-1E cells (A) INS-1E cells transduced to express either nonsense shRNA (shCtrl) or shRNA that specifically targets EDEM1 (shEDEM1) were seeded 48h before stimulation with glucose. After starvation in RPMI1640 medium without glucose for 1h, cells were incubated with 2.8mM, 14mM and 28mM glucose for 24h. Lysates and media were separated by SDS-PAGE and immunoblotted with indicated antibodies. (B) INS-1E-shCtrl and INS-1E-shEDEM1 were seeded 72h in RPMI medium with 11.1 mM glucose and insulin secretion was analyzed by ELISA assay. (C and D) For EDEM1 rescue, shEDEM1 silenced cells were transfected with EDEM1 for 48h. Cells were lysed and analyzed by Western Blotting (D) As in (C) but expression level of proinsulin and insulin was analyzed by ELISA assay. Data are representative of three independent experiments. Data are represented as mean ± SEM.

In order to determine the capacity of INS-1E cells to specifically respond to glucose, we investigated the effect of EDEM1 in glucose-stimulated insulin secretion. INS-1E-EDEM1 cells and control cells were starved for 1h in medium without glucose and then exposed to 1.6mM and 16.7 mM glucose, respectively, for 1h. Insulin secretion was determined by ELISA assay and the results showed that glucose-stimulated insulin secretion was significantly improved in the presence of overexpressed EDEM1 at stimulating (16.7mM glucose) and basal glucose concentration (1.7mM glucose) (Figure 1E). Conversely, we evaluated the consequence of silencing EDEM1 expression for proinsulin level. To deplete EDEM1, we transduced INS-1E cells with a short hairpin RNA (shEDEM1) generating the cell line INS-1E-shEDEM1. The Western blot analysis (Figure 2A) revealed a dramatic decrease in the intra- and extra-cellular proinsulin and insulin levels compared to the control cells created using scrambled sh (Western blot quantitation in Figure S2A). The ELISA assay validated the significant decrease of insulin secretion in EDEM1 depleted cells (Figure 2B). To establish the specificity of the silencing effect we transfected INS-1E-shEDEM1 cells with EDEM1 targeting its recovery (Figure 2C). Indeed, the EDEM1 level was re-established and even increased, but Western blotting analysis showed only a trend of proinsulin increase (Figure 2C, quantitation in Figure S2B). Nonetheless, we detected an enrichment in the insulin and proinsulin level by the more sensitive ELISA assay (Figure 2D), further confirmed by an increase in the INS2 mRNA (Figure S2C). The limitations of the rescuing approach could be due to the transitory effect of the transfected EDEM1 in a preconditioned silenced cell line or to unidentified post-ER consequences of insulin silencing.

### EDEM1 promotes insulin gene transcription

**Figure 3:**
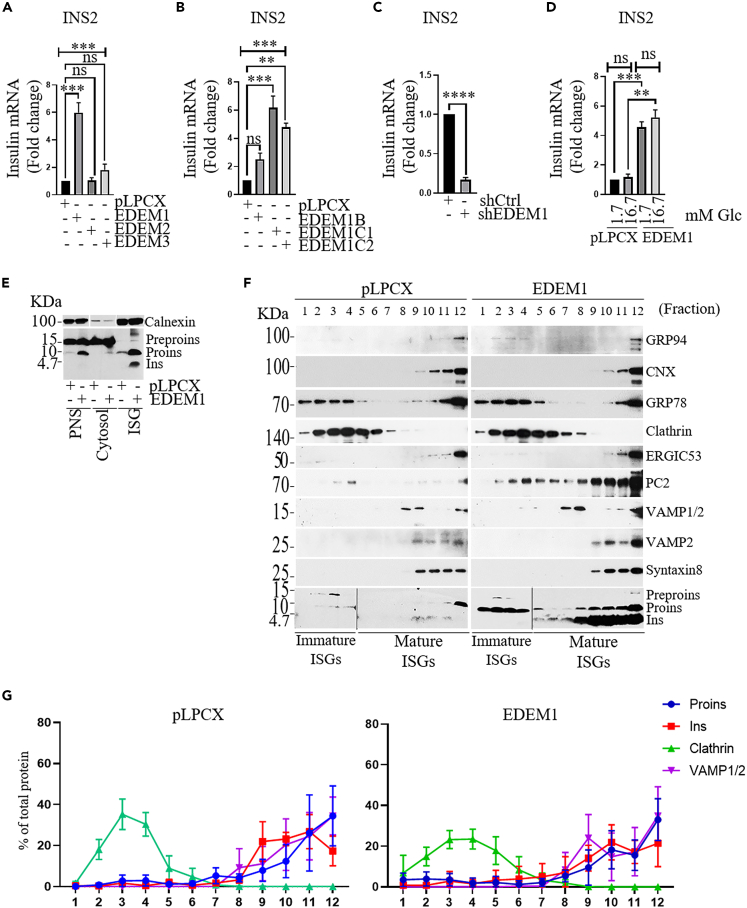
EDEM1 overexpression increases insulin mRNA and consequently leads to an increased level of properly processed and matured proinsulin (A) INS-1E-pLPCX and cells stably expressing EDEM1, EDEM2 and EDEM3 were grown in RPMI-1640 medium with 11.2mM glucose for 72h and insulin mRNA level was determined by RT PCR. (B) As in (A), but INS-1E-pLPCX cells, heterogeneous bulk cultures (EDEM1B) and two cell clones (EDEM1C1 and EDEM1C2) expressing different amount of EDEM1 protein were analyzed. (C) As in (A), but INS-1E-shCtrl and INS-1E-shEDEM1 were used. (D) INS-1E-pLPCX and INS-1E-EDEM1 cells were seeded for 72h, starved in glucose-free medium for 1h and then incubated in medium with 1.7mM and 16.7 mM glucose for 1h and insulin mRNA level was analyzed by RT-PCR. (E) INS-1E-pLPCX and INS-1E-EDEM1 cells were grown in RPMI 1640 medium with 11.2mM glucose for 72h before being subjected to the subcellular fractionation by differential centrifugation and PNS, cytosol and ISG fractions were analyzed by Western blot with anti-insulin and anti-calnexin antibodies. (F) ISGs were separated by ultracentrifugation and analyzed on a discontinuous Nycodenz gradient composed of six layers (4.4%, 6%, 8.8%,11,7%,18% and 23,4%). Fractions were separated by SDS-PAGE and characterized with markers for ER (GRP78, GRP94), ER-Golgi intermediate compartment (ERGIC) (ERGIC53), clathrin vesicles (clathrin), secretory granules (syntaxin 8, VAMP1/2, VAMP2, PC2). (G) Proteins bands from Western Blot were analyzed and quantified with ImageJ analysis software. Data are represented as mean ± SEM.

Next, we analyzed whether the overexpression of EDEM1, EDEM2, and EDEM3 proteins modulates the expression level of insulin mRNA. RT-PCR assay performed revealed increased insulin mRNA level in the presence of EDEM1, but not in the presence of EDEM2 or EDEM3 (Figure 3A. Furthermore, RT-PCR (reverse-transcriptase polymerase chain reaction) analysis in bulk and two cell clones overexpressing EDEM1 showed an increased level of insulin mRNA in all cell clones tested (Figure 3B). On the other hand, EDEM1 silencing resulted in a decrease in the insulin mRNA level (Figure 3C). The modification of the insulin mRNA during the 1h stimulation assay was not significant, as opposed to the increase found in INS-1E cells overexpressing EDEM1 (Figure 3D). These data show that overexpression of EDEM1 causes an increase in insulin mRNA leading to a rise in the polypeptide synthesis.

We further examined whether the high level of proinsulin in EDEM1 cells could alters the intracellular trafficking of proinsulin toward the secretory granules. Thus, we have separated the cytosol from the membrane fraction including the insulin secretory granules (ISGs) by differential centrifugation (Figures 3E and 3F) and further enriched the ISG fraction by Nycodenz gradient fractionation. The light fractions displayed proinsulin in chlatrin immature ISGs and the dense fractions containing mature ISGs together with syntaxin 8, PC2, and VAMP 1/2 contained mostly insulin together with some proinsulin (Figure 3F). The insulin traffic was similar in the two cell lines despite the increased amount of proinsulin synthesized in INS-1E-EDEM cells, indicating that proinsulin is efficiently released from the ER and converted into insulin in the secretory granules in the presence of EDEM1. Indeed, confocal immunofluorescence experiments showed that in the presence of EDEM1 very little proinsulin was found in the ER and no co-localization of proinsulin with calnexin or EDEM1 was observed, indicating at the same time that this increased amount of proinsulin was correctly processed in the ER and trafficked to insulin secretory granules near to the plasma membrane (Figure S3). Resuming the transport between the ER and Golgi (blocked by Brefeldin A (Bref.A)^27^) by removing Brefeldin A, revealed a rapid recovery in INS-1E-EDEM1 cells with a vesicular proinsulin/insulin at the vicinity of the membrane, as opposed to a very slow recovery in empty vector-transduced cells (INS-1E-pLPCX cells) (Figure S4). This finding prompted us to further ask whether EDEM1 may have a direct role on proinsulin folding within the ER and whether EDEM1 associates with it. Proinsulin co-precipitated with EDEM1 in INS-1E-EDEM1 cells, but not in INS1-E cells and INS-1E-shEDEM1 cells (Figure S3). Interestingly, EDEM1 was co-immunoprecipitated by GRP94 and BiP in cells overexpressing EDEM1, while no interaction was found in INS-1E cells with depleted EDEM1. Therefore, EDEM1 may act within the ER as a chaperone for proinsulin, able to associate with proteins involved in protein folding and quality control such us calnexin and GRP94,^26^ and also with the ERAD components,^12^^,^^28^ BiP, SEL1L.

Taken together, the data show that EDEM1 overexpression or silencing impacts insulin synthesis. EDEM1 could increase insulin synthesis level while conferring the INS-1E cells a specific and robust response by secreting insulin to increased glucose concentrations in the media. At the same time, this increased amount of proinsulin was correctly processed within the ER and trafficked to granules near the plasma membrane. Proinsulin traffic re-initiated after Brefeldin A interruption was restored faster in the presence of EDEM1, indicating that EDEM1 is required for the proinsulin processing within the ER.

### Insulin expression is increased in human pancreatic islets transduced with EDEM1 lentivirus

**Figure 4:**
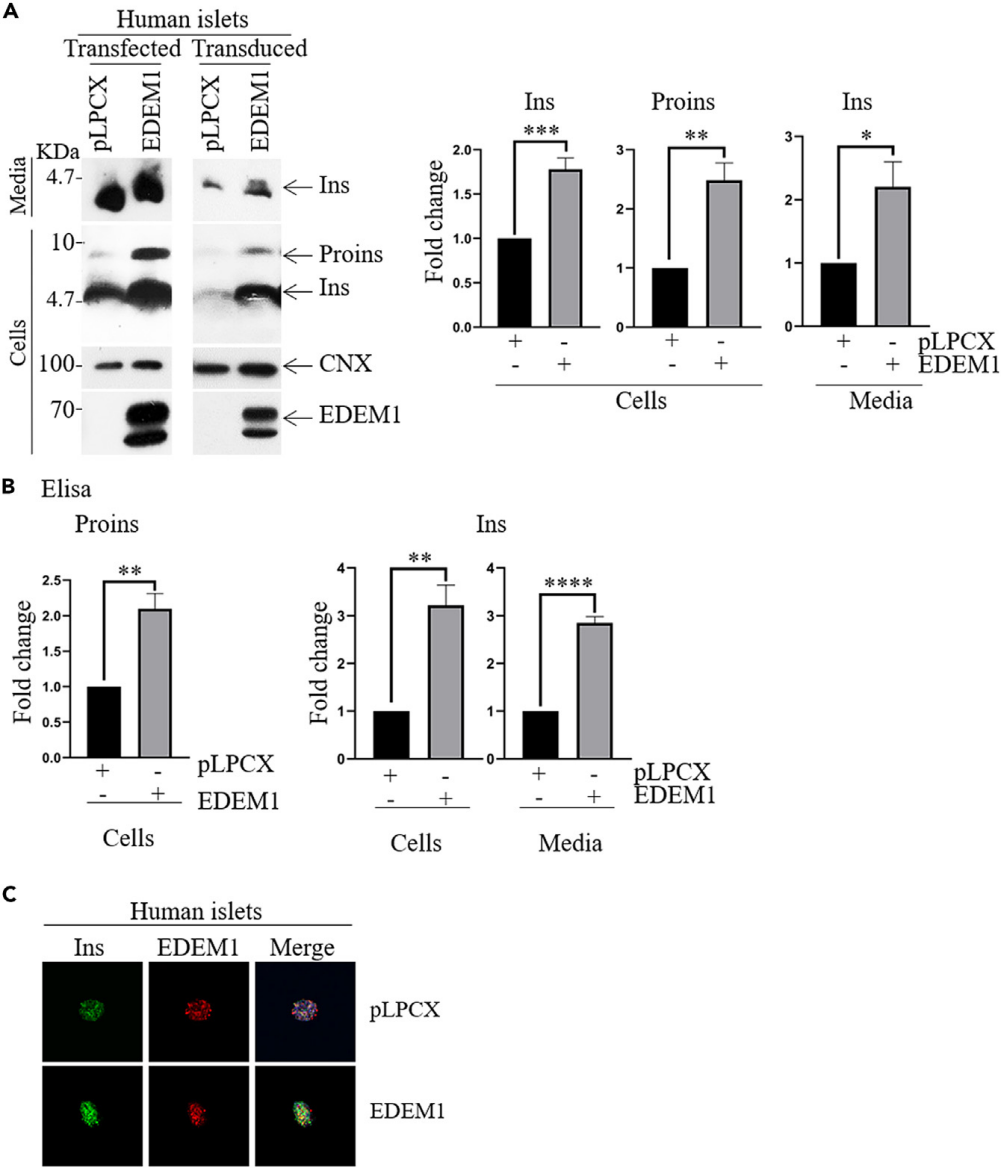
Transduction of EDEM1 in human primary pancreatic islets increases insulin content and secretion (A) Intact islets were transfected with pCMV-SPORT2-EDEM1 and transduced with lentiviruses encoding EDEM1and compared with islets transfected and transduced with empty vector (Ctrl). The islets were pre-incubated overnight in 2.8mM glucose and the next morning stimulated with 14mM glucose for 24h. Cell lysates and medium were analyzed by Western Blotting to determine insulin and EDEM1 expression. Data representative of 5 independent experiments. *Right panel* Proinsulin and insulin bands from Western Blotting were quantified. (B) As in (A) but proinsulin and insulin content and secretion were measured by ELISA assay. The insulin secretion was normalized to the total protein level. Data are represented as mean ± SEM. (C) Confocal immunofluorescence microscopy of intact islets co-stained for insulin B (green), EDEM1 (red) and DAPI (blue).

We further extended our investigations to human primary pancreatic islets. We used intact islets isolated from donors that were transduced with EDEM1 using a lentiviral system (Figure 4A, right panel), or transfected with EDEM1 (left panel) and analyzed the level of proinsulin and insulin content and insulin secretion upon 24h glucose stimulation. Overexpression of EDEM1 resulted in an increase in the steady-state level of intracellular proinsulin and insulin simultaneously with a higher secretion of insulin (Figure 4A). Quantification of intracellular proinsulin and intra- and extra-cellular insulin levels by Western blot is represented in Figure 4 the *right panel*. Further, the ELISA assay confirmed the increased proinsulin and insulin content and secretion in islets with EDEM1 overexpressed (Figure 4B). Although there is a slight difference in the insulin level determined by Western blot versus the ELISA assay, which is probably due to the different antibodies used, a significant increase in insulin synthesis and secretion upon glucose stimulation was determined by both methods in the presence of EDEM1. Immunofluorescence microscopy revealed the presence of endogenous EDEM1 in islets and overexpressed EDEM1 in transduced islets while confirming the improved insulin expression in the latter (Figure 4C). These data show that similar to the INS-1E cells results, EDEM1 strongly influences insulin secretion in intact islets.

### Increased glucose tolerance in a chemically induced diabetic model in rat

**Figure 5:**
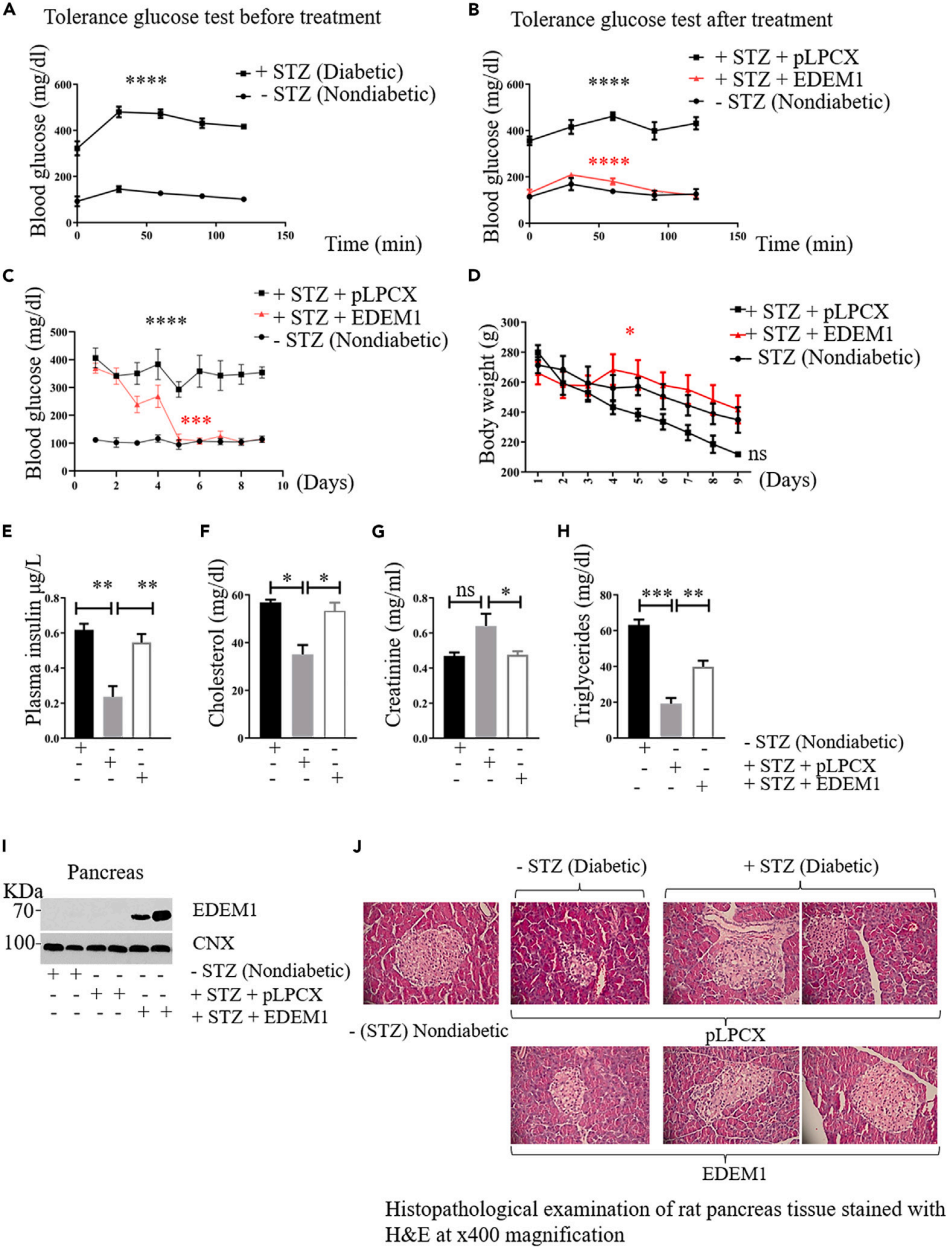
The effect of EDEM1 on insulin secretion level in STZ-diabetic rats (A) Glucose tolerance test was measured before (p < 0.0001) and after treatment (p < 0.0001) with EDEM1-lentivirus in STZ-diabetic rats compared with healthy animals. Glucose (1g glucose/kg rat) was administered to nondiabetic rats, to diabetic rats and to EDEM1-treated rats. The glucose level was examined before glucose loading (A), and at 0, 30, 60, 90 and 120 min after glucose loading. (B)*.* Every point represents a mean of values in 4 nondiabetic rats, 4 diabetic rats and 9 treated rats. Animals were sacrificed after 9 days of treatment. (C) Fasting blood glucose levels were daily determined starting with the first day post-STZ-treatment until the last day of treatment. Results are represented as daily total mean values (p < 0,0005). (D and E) *Body weight* (E) Plasma insulin concentrations were determined by ELISA assay. Values are expressed as mean ± SEM. p < 0.002 versus diabetic rats. (F–H) Cholesterol (p < 0.03) (G) Creatinine (p < 0.01), (H)*.* Triglycerides (p < 0.005) were determined at the end of the experiment and body weight daily (p < 0.01). The results are expressed as the mean values ± SEM). (I) Pancreas was collected, lysed and analyzed by Western Blot using antibodies against EDEM1. (J) *Pancreas histopathology.* Normal control rat pancreas showing several Langerhans islets with dimension variability. Non-diabetic rat treated with pLPCX -top panel, Non-diabetic rat treated with EDEM1. Diabetic control rat pancreas treated with pLPCX showing small and large Langerhans islets. Diabetic rat pancreas treated with EDEM1 showing numerous small and large Langerhans islets (H&E, X400). At least three different sections per sample were examined. Notes: Data are represented as mean ± SEM(n = 5, n-7), Abbreviation: STZ, streptozotocin.

To examine the physiological role of EDEM1 in the animal model, diabetes was induced by injecting a single dose of low streptozotocin (STZ) concentration in Wistar rats, as previously described.^30^ We used this approach to induce rapid hyperglycaemia and diabetes mellitus, and while this model is not ideal, we were able to obtain diabetic response in animals that lost less than 20% of their islets in 48h, the starting point of the treatment experiment. We therefore tested whether gene therapy using EDEM1 lentivirus injections every 24h could increase the insulin secretion and rescue the hyperglycemic response of these rats. We performed a glucose tolerance test that revealed a decreased glucose tolerance in the diabetic group, as compared with the healthy group (Figure 5A). Importantly, in the 9^th^ day of treatment with EDEM1 lentivirus, the diabetic and EDEM1 treated group showed an increased glucose tolerance that was similar to the healthy rats group, as opposed to the diabetic rats that received empty vector as treatment (Figure 5B). Moreover, treatment with EDEM1 resulted in a significant decrease in blood glucose level starting with the second day of treatment and reaching values close to the nondiabetic group (around 100 mg/dl) at the end of the experiment. By contrast, blood glucose levels of diabetic group were above 300 mg/dl during the experiment (Figure 5C). Body weight measurements indicated a protective effect of EDEM1 on STZ-induced body weight loss (Figure 5D). We found that serum insulin levels were significantly higher in the group treated with EDEM1 compared with the diabetic group and were similar to the nondiabetic group (Figure 5E). The biochemical data also indicate that the pancreas function of diabetic rats has been improved by EDEM1 administration and there is a protective role of EDEM1 on the liver function (Figures 5F, 5G and 5H). Western blot of the resected pancreas demonstrated increased level of EDEM1 in the treated rats, confirming the efficiency of the procedure (Figure 5I).Histopathological observations of the pancreas were performed using haematoxylin and eosin (H&E) staining (Figure 5J). In the control (healthy non-diabetic untreated rat)-we noticed that Langerhans islets were mostly roundish, or round to oval, well delineated and dual population of cells present. All the insular cells had more eosinophilic cytoplasm, without ballooning degeneration and smaller nuclei without nuclear pleomorphism. In diabetic rats treated with empty vector – all Langerhans islets (both small and large) were mostly roundish, well delineated, with two cell population: polygonal medium-sized cells within the center (these cells had hyperchromatic central nuclei, focally mild pleomorphic and pale, slightly granular cytoplasm with occasional ballooning degeneration) and small central nuclei. In the pancreas of EDEM1 treated diabetic rats– we found frequent Langerhans islets with variable size, mostly roundish, with two cell populations: rare polygonal medium-sized cells with occasional ballooning degeneration and hyperchromatic central nuclei and peripheral-small with granular eosinophilic cytoplasm and small central nuclei. It is important to mention the fact that the overexpression of EDEM1 or empty vector in rats not treated with streptozotocin did not cause changes in the number of islets of Langerhans and body weight (Table S3). The results of these investigations suggest that the healthy islets of diabetic rats treated with EDEM1 were able to increase the production and secretion of insulin and to overcome the decreased islets number.

### EDEM1 suppresses IRE1/JNK/c-Jun signaling pathway

ER stress and the unfolded protein response (UPR) have a crucial role in the function of the β−pancreatic cells and diabetes. Under hyperglycemic conditions, the UPR is hyperactivated leading to IRE1 phosphorylation, XBP1 mRNA splicing and consequently insulin mRNA degradation. The IRE1-XBP1s pathway also regulates the efficient folding of proinsulin.^31^^,^^32^ To understand whether EDEM1 overexpression can modulate ER stress, we examined the expression level of the most important stress sensor proteins as response to the treatment of cells with chemical stressors (DTT (DL-dithiothreitol), tunicamycin (Tuni), thapsigargin (Tg) and kifunensin (Kif.)). Western blot experiments showed an inhibition of the IRE1 pathway via P-IRE1-IRE1-CHOP and an activation of the ATF6 pathway^33^ via chaperones and ERAD proteins compared with INS-1E-pLPCX cells. We found increased expression level of ER folding chaperones (BiP and GRP94) and ERAD components (SEL1L, XTP3B, Derlin1). In the presence of DTT, there was an increase in P-IRE1, P-PERK and CHOP in INS-1E-pLPCX cells that was significantly reduced in the presence of EDEM1, which may suggest an adaptative UPR activation in the presence of EDEM1 and an apoptotic UPR activation in control cells (Figures S6 and S7A). Important to note is that treating cells with any of these chemical stressors resulted in a protective effect on the expression level of proinsulin and insulin in the presence of EDEM1 compared to control (Figure S6A).

**Figure 6:**
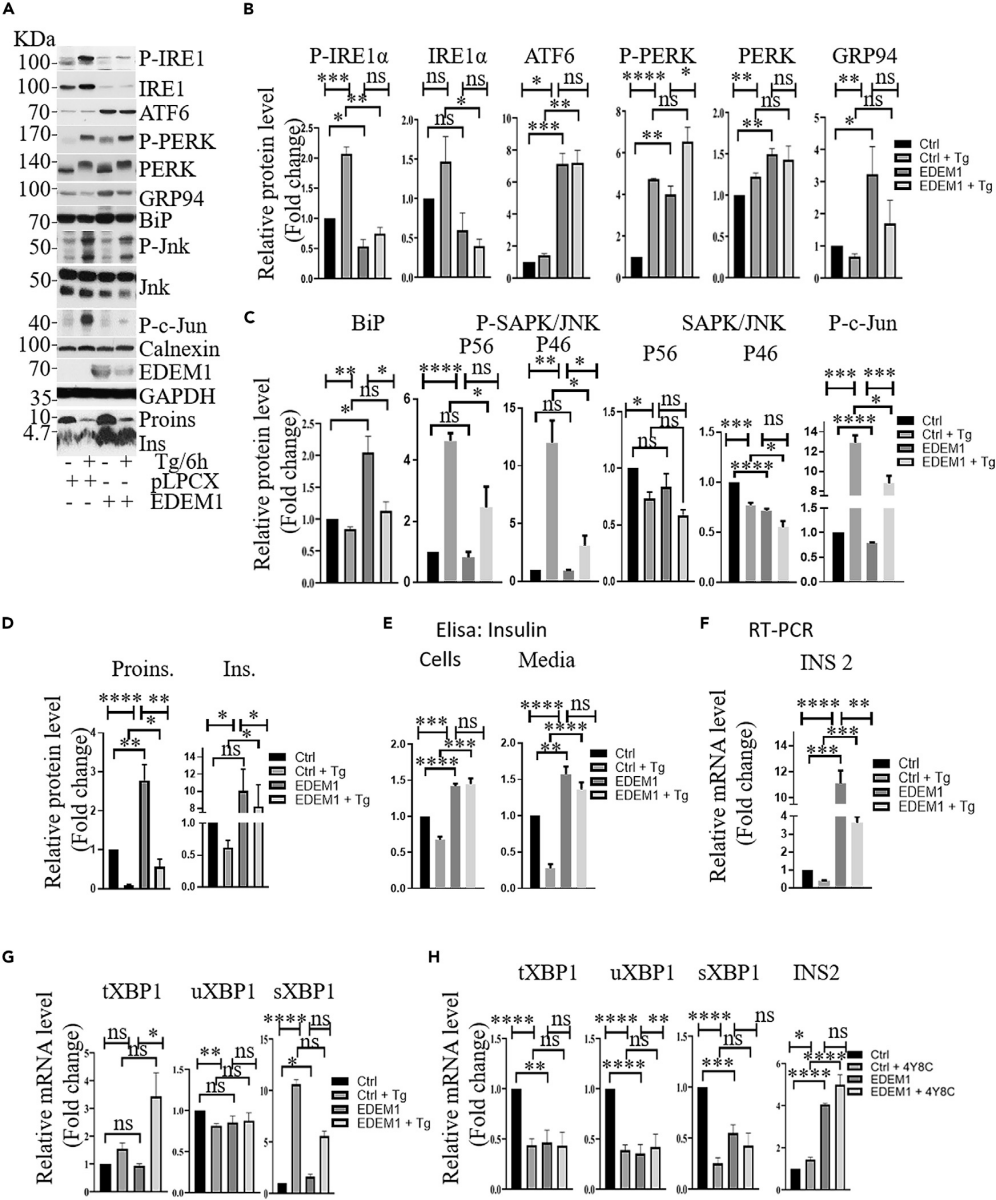
EDEM1 modulates ER stress in pancreatic beta cells treated with thapsigargin (A–D) Immunoblots from lysates of INS-1E-pLPCX and INS-1E-EDEM1 cells were treated or not with thapsigargin (Tg) for 6h. Equal amounts of protein were resolved by SDS-PAGE and immunoblotted with indicated antibodies (B, C, D) Protein bands from Western Blotting were quantified using ImageJ program. (E) As in (A) but insulin was determined in cell lysates and culture medium by ELISA assay. (F) As in (A) but total RNA was isolated and performed RT-PCR to determine the level of INS2, tXBP1, uXBP1 and sXBP1. (G) As in (H) but cells were treated with 4y8C inhibitor for 24h before total RNA was isolated and performed RT-PCR to determine the level of tXBP1, uXBP1, sXBP1 and insulin mRNA. Data are representative for at least three independent experiments. Data are represented as mean ± SEM.

We further investigated the molecular mechanism by which EDEM1 can modulate the ER stress. We found that EDEM1 overexpression in thapsigargin-treated cells resulted in the inhibition of the IRE1/JNK/c-Jun signaling pathway (Figures 6A, 6B and 6C), and maintained an increased level of insulin mRNA (Figure 6D) and also an increased insulin secretion (Figure 6E). JNK signaling pathway could be involved in the regulation of many cellular events, including autophagy, proliferation or cell death, being suggested to have a pro-apoptotic or anti-apoptotic role by stimulating the expression of specific genes. In order to evaluate if EDEM1 can also affect downstream processes of ER stress, we further analyzed the expression level of LC3, BAX, ATG3 and ATG12, proteins involved in autophagy. In the same type of experiment as above, INS-1E cells treated with thapsigargin, we found that under ER stress conditions, EDEM1 overexpression decreased the rate of apoptosis by suppressing the IRE1/JNK pathway and subsequently inhibiting LC3, BAX, and ATG3 and ATG12 (Figure S7A). Moreover, we found that overexpression of EDEM1 could significantly decrease XBP1 splicing in cells treated with thapsigargin (Figure 6G). To inhibit the IRE1-XBP1 pathway, we used with the chemical inhibitor 4y8C reported to inhibit RNase activity of IRE1 and lower blood glucose levels and increases serum insulin levels in diabetic animals.^34^ We performed RT-PCR to quantify the level of tXBP1, usXBP1 and sXBP1 and found that the treatment of cells with 4y8C led to the decrease of XBP1 splicing and it is interesting to note that EDEM1 overexpression could mimic the effect of this inhibitor (Figure 6H). The inhibition of the IRE1-XBP1 pathway by the overexpression of EDEM1 may have a protective role on insulin, thus decreasing the rate of insulin mRNA degradation.

It has been reported that there are differences between the stressors induced ER stress versus the physiological stress of the β-cell induced upon glucose stimulation.^17^ Treatment of beta cells with high levels of glucose hyperactivates IRE1 and decreased insulin mRNA level.^35^ Accordingly, we further examined whether EDEM1 has a protective effect against ER stress induced by glucose-stimulated insulin secretion. High glucose (14 and 28mM) treatment induced the expression of P-IRE1 and P-PERK, and the expression of these proteins was increased by EDEM1 overexpression (Figure S6E). Increased expression level of ER chaperones such BiP and GRP94, ER stress markers and also insulin expression level may suggest that EDEM1 can restore endoplasmic reticulum homeostasis and promote β-cell adaptation. The significant decrease in LC3BI/II and P62 accumulation in the presence of EDEM1 are indicators that EDEM1 could help in clearing protein aggregates by accelerating ER-phagy (Figure S6E)^36^. These data show that EDEM1 protects the INS-1E cells from the chemically induced stress modulating two pathways involving IRE1 activation, IRE1/XBP1s and IRE1/JNK/c-Jun.

**Figure 7:**
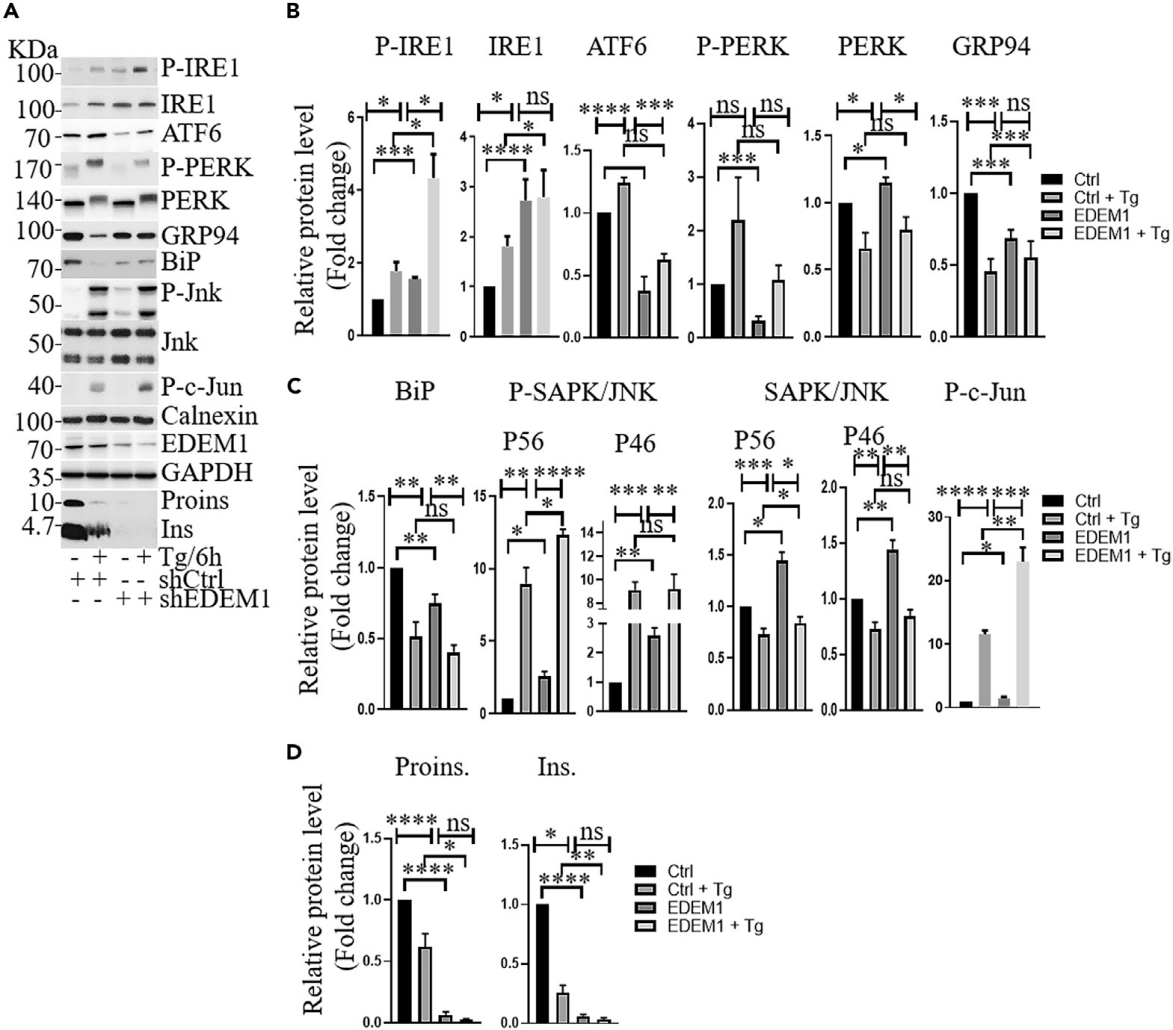
EDEM1 silencing activates the IRE1/JNK/c-Jun signaling pathway and decreases insulin mRNA in INS-1E cells treated with thapsigargin (A) INS-1E-shCtrl vs. INS-1E-shEDEM1 cells were treated or not with thapsigargin for 6h. Cell lysates were analyzed by Western Blotting with indicated antibodies. (B–D) Protein bands from Western Blotting were quantified using ImageJ program. Data are representative for at least three independent experiments. Data are represented as mean ± SEM.

To validate the role of EDEM1 in inhibiting the IRE1/JNK/c-Jun signaling pathway, we also treated INS-1E-shCtrl and INS-1E-shEDM1 cells with thapsigargin for 6h. The results disclosed that the treatment of cells with thapsigargin induces an activation of the IRE1/JNK/C-JUN pathway (Figure 7) and causes apoptosis through the consequent induction of the LC3, BAX, and ATG3 and ATG12 (Figure S7) and EDEM1 silencing potentiates this effect. Thus, the expression level of EDEM1 can modulate the ER stress and insulin transcription level in pancreatic beta cells. We show here that EDEM1 impacts the ER stress via IRE1-XBP pathway, inhibiting insulin mRNA degradation and IRE1/JNK/c-Jun signaling pathway, which in turn suppresses the insulin mRNA.

## Discussion

The most important finding of this article is that overexpression of EDEM1, the key factor of the ER quality control process that selects the misfolded proteins destined for degradation, markedly increases the insulin secretion stimulated with glucose in rat and human β-pancreatic cells. The efficient insulin release is supported by the synthesis of a higher amount of insulin that is stored in the granules of these EDEM1 overexpressing cells.

To investigate the events leading to the increased insulin content within these cells, we determined the amount of proinsulin level at steady state and found that besides insulin, the proinsulin content was also higher in most of the EDEM1 containing clones of INS1-E cells stably transduced with EDEM1. Importantly, the effect of EDEM1 is reversed upon EDEM1 silencing, as shown by the analysis of the EDEM1 knocked down INS-1E cells. The down regulation of proinsulin and insulin content, as well as insulin secretion in INS-1E-shEDEM1 cells confirms the specific role of EDEM1 on insulin expression level. The fact that EDEM1 induced an increase in the ER chaperones BiP and GRP94 and was found in complex with these two chaperones and with proinsulin, indicates that in concert with the BiP and GRP94, previously reported to chaperone proinsulin, EDEM1 regulates proinsulin stability within the ER. We have recently reported that EDEM1 is part of multiple complexes within the ER networking with the folding chaperones, but also with the ERAD machinery and the autophagy proteins.^4^ The significant decrease in LC3B-II/I, BAX and ATG3, ATG12 in the presence of EDEM1 are indicators that EDEM1 could help in clearing protein aggregates by accelerating ER-phagy.^36^ We should however note that EDEM2, the key EDEM protein that initiates the ERAD,^37^ impairs insulin synthesis, thus arguing against a role of EDEM proteins in the ERAD of β-cells, but further investigations are needed to elucidate whether EDEM1 and EDEM2 play different roles in these specialized cells.

Human pancreatic islets transduced with EDEM1 respond to glucose-stimulation by secreting approximately three times more insulin, indicating a similar effect as found in INS-1E-EDEM1 cells. Similar to rat pancreatic cells, human islets overexpressing EDEM1 display an increase in intracellular insulin levels and secreted insulin in response to glucose stimulation. Moreover, diabetic rats treated with streptozotocin and transduced with EDEM1 produce enough insulin to maintain glucose homeostasis at a level comparable with healthy rats. Normoglycemia was obtained in the serum of these rats within 5 days of treatment with EDEM1. We show that treated rats revealed a 3-fold increase in insulin secretion. Insulin has important effects on key steps in the metabolism of lipids and lipoproteins, and alterations in lipid metabolism are common in diabetic population. In the present study, streptozotocin-induced abnormalities in blood glucose, TG, cholesterol and the other lipid abnormalities developed in STZ-diabetic rats were countered by treatment with EDEM1. Histopathological examination of rat pancreas tissue showed that overexpression of EDEM1 in animal model does not influence the total number and the dimensions of Langerhans islets. Streptozotocin-induced diabetic rats treated with EDEM1 show decreased glycemia values, increased glucose tolerance and higher serum insulin.

Several studies reported that insulin gene transcription is down-regulated in diabetic animals, thus impairing proinsulin and insulin expression. The EDEM1 transduced cells show a clear increase in the proinsulin mRNA abundance, as compared to control INS-1E cells and this indicates a new role for EDEM1 in the regulation of insulin transcription. Further validation came from the knock-down of the EDEM1 resulting in a dramatic decrease of the proinsulin mRNA level. Since IRE1, one of the crucial ER stress sensors for the β-pancreatic cells was reduced in the presence of EDEM1, mainly due to its degradation by overexpressed EDEM1, as previously reported,^30^ we investigated whether IRE1 could impair the insulin mRNA transcription or degradation. In the presence of thapsigargin-induced ER stress, the XBP1 splicing of EDEM1 cells was reduced. Together with the fact that an inhibitor of IRE1 RNase activity reduced the splicing effect of IRE1 on XBP1 mRNA, we conclude that EDEM1 inhibits the IRE1-XBP1 pathway. IRE1 activation during ER stress triggers a specific endoribonuclease activity and initiates the splicing of the mRNA encoding XBP1s and ER-targeted mRNAs, including insulin.^35^ Thus, the inactivation of the IRE1 endoribonuclease activity reduces the insulin mRNA degradation protecting the beta cell. Activation of IRE1 may also affect another signaling pathway, i.e., IRE1/JNK/c-Jun, reported to suppress the insulin gene transcription.^36^ Our data show that during thapsigargin -induced ER stress, there is a reduced level of phosphorylated active IRE1, JNK and c-Jun in the presence of EDEM1, revealing the inhibition of the IRE1/JNK/c-Jun signaling. Thus, we provide evidence that EDEM1 increases the insulin protein level and mRNA level, and protects pancreatic β-cells during ER stress suppressing the IRE1/JNK/c-Jun signaling pathway.

In conclusion, our results show that EDEM1 protein has an important role in regulating insulin gene expression and insulin secretion, inhibiting the IRE1 endonuclease activity and IRE1/JNK/c-Jun signaling pathway. The mechanism of action is not fully elucidated, but involves reducing ER stress via the IRE1/JNK/c-Jun pathway, and consequently preventing insulin mRNA degradation by IRE1 activation and inhibition of P-*c*-Jun, which suppresses the insulin gene expression. These data support EDEM1 as a potential target for the treatment of diabetes.

### Limitations of the study

The experiments including human islets have been limited to the determination of the intracellular insulin content and secretion in the presence of EDEM1 due to the human islets supply constraints. While finding a beneficial effect of EDEM1 treatment upon insulin secretion and glucose tolerance in rats, our data cannot distinguish between a direct or indirect effect of EDEM1.

## STAR★Methods

### Key resources table

**Table:** 

REAGENT or RESOURCE	SOURCE		IDENTIFIER
**Antibodies**			

Insulin B (C-12)	Santa Cruz Biotechnology		Cat# sc-377071, RRID:AB_2800506
Insulin (H-86)	Santa Cruz Biotechnology		Cat# sc-9168, RRID:AB_2126540
C-peptide [1H8]	Abcam		Cat# ab8297, RRID:AB_306447
EDEM1	SIGMA-ALDRICH		Cat# E8406, RRID:AB_1078720
Beta-Actin	Abcam		Cat# ab8226, RRID:AB_306371
Glut2 (C-19)	Santa Cruz Biotechnology		Cat# sc-7580, RRID:AB_641066
PC2 (H-20)	Santa Cruz Biotechnology		Cat#sc-22891, RRID:AB_2251901
Vamp2 (3E5)	Santa Cruz Biotechnology		Cat# sc-69706, RRID:AB_2212614
Vamp 1/2 (3H3117)	Santa Cruz Biotechnology		Cat# sc-73249, RRID:AB_1129807
GRP78 (A-10)	Santa Cruz Biotechnology		Cat# sc-376768, RRID:AB_2819145
GRP94 (H-10)	Santa Cruz Biotechnology		Cat# sc-393402, RRID:AB_2892568
Phospho-IRE1alpha (Ser724)	ThermoFisher SCIENTIFIC		Cat# PA1-16927, RRID:AB_2262241
IRE1α (14C10)	Cell Signaling		Cat# 3294, RRID:AB_823545
Phospho-PERK-T980 (16F8)	Cell Signaling		Cat# 3179, RRID:AB_2095853
PERK (C33E10)	Cell Signaling		Cat##3192, RRID:AB_2095847
ATF6	Novus Biologicals		Cat# 40256, RRID:AB_2058775
P-eiF2alpha (S51)	Cell Signaling		Cat# 9721S, RRID:AB_330951
eIF2α	Cell Signaling		Cat# 9722S, RRID:AB_2230924
Tubulin	Abcam		Cat# ab6046, RRID:AB_2210370
Calnexin	Abcam		Cat# ab-22595, RRID:AB_206900
Clathrin Heavy Chain	BD Transduction Laboratories		Cat# C43820-0, 0499
GRP94(H-10)	Santa Cruz Biotechnology		Cat# sc-393402
Phospho-SAPK/JNK (Thr183/Tyr185) (81E11)	Cell Signaling		Cat# 4668, RRID:AB_823588
SAPK/JNK	Cell Signaling		Cat# 9252, RRID:AB_2250373
Phospho-c-Jun (Ser73) (D47G9)	Cell Signaling		Cat##3270, RRID:AB_2129575
Syntaxin 8	BD Transduction Laboratories		Cat# 611352, RRID:AB_398874
LC3 (5H3)	nanoTools		Cat# 0231/100, RRID:AB_2722733
P62	Cell Signaling		Cat# 5114, RRID:AB_10624872
P-ERK	Cell Signaling		Cat# 4370P
ERK	Cell Signaling		Cat# 4695P
ATF-4 (D4B8)	Cell Signaling		Cat#11815RRID:AB_2616025
BAX	ThermoFisher SCIENTIFIC		Cat# MA5-14003, RRID:AB_10979735
ATG3	Cell Signaling		Cat# 3415, RRID:AB_2059244
ATG12 (D88H11)	Cell Signaling		Cat# 4180, RRID:AB_1903898
Mouse anti-rabbit IgG-HRP	Santa Cruz Biotechnology		Cat# sc-2357, RRID:AB_628497
m-IgGxBP-HRP	Santa Cruz Biotechnology		Cat# sc-516102, RRID:AB_2687626
mouse anti-goat IgG-HRP	Santa Cruz Biotechnology		Cat# sc-2354, RRID:AB_628490

**Chemicals, peptides, and recombinant proteins**			

4y8C inhibitor	Millipore		Cat# HY-19707
Streptozotocin	Sigma-Aldrich		Cat# S0130
Kifunensine	Santa Cruz Biotechnology		Cat# sc-201364A
3-Methyladenine	Sigma-Aldrich		Cat# M9281
Histodenz	Sigma Aldrich		Cat# D2158
Tunicamycin	Santa Cruz Biotechnology		Cat# sc-3506A
Thapsigargin	Santa Cruz Biotechnology		Cat# sc-24017A
Brefeldin A	Santa Cruz Biotechnology		Cat# sc-200861
Dithiothreitol	Santa Cruz Biotechnology		Cat# sc-29089B
Glycerol	Sigma-Aldrich		Cat# G5516
Bovine Serum Albumin	Sigma-Aldrich		Cat# A9418
2-Mercaptoethanol	Sigma Aldrich		Cat# M6250
Penicillin/Streptomycin	Gibco™		Cat# 5140122
Blotto, non-fat dry milk	Santa Cruz Biotechnology		Cat# sc-2324

**Critical commercial assays**			

Rat Insulin ELISA	Mercodia Developing diagnostics		Cat.No.10-1250-01, RRID:AB_2811229
Rat/Mouse Proinsulin ELISA	Mercodia Developing diagnostics		Cat.No.10-1232-01
Human Insulin ELISA	Mercodia Developing diagnostics		Cat.No.10-1113-01, RRID:AB_2877672
Human Proinsulin ELISA	Mercodia Developing diagnostics		Cat.No. 10-1118-01, RRID:AB_2754550
SensiFAST SYBR No-ROX One Step kit	Bioline		Cat.No.BIO-72005

**Deposited data**			

Original Western blots	This manuscript		Mendeley Data, V2, https://doi.org/10.17632/txxsv97xvv.2

**Experimental models: Cell lines**			

Rat: INS-1E cells (male)	Lonza		RRID:CVCL_0351
Human pancreatic islets (male)	Prodo Laboratories Inc.		Cat. No. PIM-S001GMP

**Experimental models: Organisms/strains**			

Wister Albino Rats (male)	University of Medicine and Pharmacy “Carol Davila”, Bucharest	N/A	

**Oligonucleotides**			

Primers sequences are listed in Table S2.	This paper	N/A	
shRNA sequences used for EDEM1	This paper	Cat.No. 336314	

**Recombinant DNA**			

cDNA of pCMVSport-EDEM1, pCMVSport-EDEM2, pCMVSport-EDEM3	Dr.K.Nagata and Dr.N.Hosokawa (Institute of Frontier Medical Science, Kyoto University	N/A	
cDNA of pLPCX-EDEM1, pLPCX-EDEM2, pLPCX-EDEM3	This paper	Clontech	

**Software and algorithms**			

Prism software (GraphPad v.6.4.3).	GraphPad		https://www.graphpad.com
Image J	Image J		https://imagej.nih.gov/ij/download.html
Endnote	Endnote		https://endnote.com

**Other**			

RPMI	Gibco		61870044
FBS	Gibco		10270106
Glutamine	Gibco		25030081
HEPES	Gibco		15630056
Sodium pyruvate	Gibco		11360039
Penicillin/Streptomycin	Gibco		15140122
Protease inhibitors	Roche		11697498001

### Resource availability

#### Lead contact

Further information and requests for resources and reagents should be directed to and will be fulfilled by the lead contact: Stefana M. Petrescu (stefana.petrescu@biochim.ro).

#### Materials availability

This study did not generate new unique reagents.

### Experimental model and study participant details

*In vitro* experiments were conducted using INS-1E cell line, derived from male rat. Cells were tested for mycoplasma contamination and found to be contamination-free.

Human islets were obtained from male cadaveric donors (Prodo Laboratories Inc). The age of the donors was 23/32 and 67, with BM1 (kg/m^2^) 22.5/24.8, 30.5 and HbA_1c_ 5.3%/5.4%/5.5%. Human islets were cultured in PIM(S) media with 5.8mM glucose and supplemented with 25ml of PIM(ABS)-Human AB Serum and 5ml PIM(G)Glutathione/Glutamine.

For *in vivo* animal studies, male rats, aged between 3 and 4 weeks, were used. Rats received standard chow and water *ad libitum*. Animals were stratified by blood glucose and body weight values prior to randomization. Rats were intraperitoneally injected with freshly prepared streptozotocin (STZ) solution (60g/kg rat) in 0.1 M citrate buffer (pH 4.5). Only rats with blood glucose concentration over 250 mg/dl at 48h from the STZ injection were used in the experiment. Animals were separated into two groups: diabetic rats treated with pLPCX-EDEM1, N=9, second group, diabetic rats treated with empty vector, N=4 and non-diabetic rats as negative control. The glucose level was monitored daily for nine days, after which the rats were killed by cardiac puncture under isoflurane anaesthesia. The study protocol was approved by the Ethics Committee of the’’Carol Davila’’ University of Medicine and Pharmacy in accordance with the EU Directive 2010/63 approved in 2014.

#### Cell lines

Rat insulinoma INS-1E cells (Lonza) were cultured in RPMI 1640 medium (Gibco by Life Technologies) supplemented with 10% fetal bovine serum (FBS) (Gibco by Life Tehnologies), 1mM sodium pyruvate, 10mM HEPES, 100 units per ml penicillin, 10μg/ml streptomycin and 50μM 2-mercaptoethanol. They were maintained at 37°C and 5% CO_2_ in a humidified atmosphere. Cells were tested for mycoplasma contamination and found to be contamination-free. Human islets were obtained from cadaveric donors from Prodo Laboratories Inc. and cultured in PIM(S) media (Prodo labs-Cat no.-PIM-S001GMP) (500 ml bottle of PIM(S), which has a concentration of 5.8mM glucose and supplemented with 25ml of PIM(ABS)-Human AB Serum (Prodo labs-Cat no. PIM-ABSOO1GMP) and 5ml PIM(G)Glutathione/Glutamine (Prodo labs-Cat no.-PIM-G001GMP).

### Method details

#### Generation of stable cell lines

INS-1E cells were stably transfected to express heterologous EDEM1, EDEM2 and EDEM3 or empty vector (pLPCX) using a retroviral system. The cDNA of EDEM1, EDEM2 and EDEM3 cloned into the pCMVSport were recloned into the pLPCX retroviral vector (Clontech). The plasmids obtained were sequenced and then transfected together with packaging plasmid pCL-Ampho (Imgenex) into HEK293T cells to obtain lentiviral particles. In parallel, a control cell line was built containing empty vector-INS-1E-pLPCX. Transduced cells were selected using puromycin and expression level of EDEM1, EDEM2 and EDEM3 was measured by Western Blotting. For the EDEM1 silencing, INS-1E cells were transduced with four different shRNA sequences for rat EDEM1 and one negative control shRNA, as provided by Qiagen (Cat. No.336314). The best silencing obtained with the shRNA sequence, TCTCGtcaacgatgtactaggaaattCTTCCTGTCAaatttcctagtacatcgttgaCT, was further used throughout this study. Bulk cells were cloned by serial dilution and expanded in medium containing puromycin. The shRNA sequences used for EDEM1 silencing in INS-1E cells are listed in Table S1.

#### Glucose-stimulated insulin secretion

Cells seeded in equal numbers were preincubated for 1 hour in Krebs-Ringers-Bicarbonate-HEPES buffer supplemented with 0.5% bovine serum albumin and without glucose. After preincubation, cells were incubated in the same buffer plus the different glucose concentrations (1.67 mM and 16.7 mM glucose) for 1h. For overnight insulin secretion, cells were incubated for 1h in RPMI1 medium without glucose followed by 24h incubation in RPMI medium with 2.8 mM, 14 mM and 28 mM glucose. Insulin secretion was normalized to the total protein concentration determined by BCA assay. Insulin and proinsulin content and secretion were analysed by ELISA, Western Blotting and Immunofluorescence assays.

#### Western blot analysis

Cells were lysed on ice with RIPA buffer (25mM Tris-HCl, 1% Triton X-100, 1% sodium deoxycholate, 0.1% SDS, 150 mM NaCl, pH 7.6) supplemented with protease and phosphatase inhibitor cocktail and NEM (N-ethylmaleimide). Human islets were lysed on ice with Tris-EDTA buffer (10 mM Tris-HCl, 1mM disodium EDTA, pH 8.0) supplemented with 1% Triton X-100 and protease inhibitor cocktail. The pancreas was lysed in RIPA buffer supplemented with protease inhibitor cocktail. Equal amounts of total protein were loaded on Tris/Glycine and Tris-Tricine gels and visualized by immunoblotting with specific antibodies.

#### RNA extraction and real-time PCR analysis

Total RNA was isolated using TRIzol reagent (Invitrogen) according to the manufacturer’s protocol. RNA concentration was measured using an NanoDrop (Thermo Scientific) and equal amounts of total RNA were used. Quantitative PCR assays were conducted using the SensiFAST SYBR No-ROX One Step kit (Cat.No.BIO-72005, Bioline), according to the manufacturer’s protocol. For inhibition of IRE1 activity, cells were cultivated in RPMI1640 medium for 48h and then treated with 50μM 4y8C for 24h, an IRE1 inhibitor with blocks substrate (RIDD) access to the active site of IRE1 and inactivates XBP1 splicing and IRE1 mediated mRNA degradation.^38^ The primers sequences used to amplify Rat Insulin II, total-XBP1, unspliced XBP1, spliced-XBP1, β-actin are provided in Table S2. Quantitative PCR was carried out using the Corbett Rotor Gene 6000 real-time PCR system using the following cycling conditions: 45°C for 10 min, 95°C for 2 min, followed by 40 cycles at 95°C for 5s, 60°C for 10s and 72°C for 5sec. Relative mRNA expression was normalized to the β-actin level.

#### Immunofluorescence and confocal microscopy

INS-1E cells and human islets were seeded onto chamber slides and allowed to attach for 48h, then stimulated with 14mM glucose for 24h. Cells were washed with PBS and fixed with 4% PFA (paraformaldehyde), permeabilised in PBS containing 0.2% Triton-X-100 and blocked with 0.5% BSA in PBS. Incubation with primary antibodies was performed for 30 minutes at room temperature, followed by washings and incubation with specific secondary antibodies conjugated with Texas Red or FITC. Nuclei were stained with DAPI (4′,6-diamidino-2-phenylindole dihydrochloride) and samples were examined with an LSM710 confocal microscope and Zen2010 software (Carl Zeiss).

#### Subcellular fractionation for insulin secretory granules enrichment

INS-1E-pLPCX and INS-1E-EDEM1 cells were seeded 48h before stimulation with glucose. After starvation in RPMI 1640 medium without glucose for 1h, cells were stimulated in medium with 11,2mM glucose for 24h, washed and scraped in PBS and then re-suspended in 0.27 M sucrose, 10 mM MOPS-Tris (pH 6.8) (SMT buffer) by three strokes through a 21-gauge needle followed by three strokes through a 25-gauge needle. Cell debris and nuclei were removed and supernatant was transferred to a new tube and the remaining pellet was re-suspended in SMT by three strokes through a 21-gauge needle followed by three strokes through a 25-gauge needle and then centrifuged for 5 min al 1000xg. Pooled supernatants were centrifuged for 10 min at 1000xg to generate the postnuclear supernatant (PNS). To separate organelles from the cytosol, PNS was centrifuged at 24700xg for 20 min resulting two fractions, the cytosol and the ISG pellet. The ISG pellet was re-suspended in SMT buffer and loaded on a discontinuous Nycodenz gradient and centrifuged at 107000xg for 75 min^39^^,^^40^ for further fractionation of the granules. The resulting fractions were analysed by Western blot to identify immature and mature ISGs.

#### Animals

Three-four-week-old male Wistar Albino rats from Animal Husbandry of ’’Carol Davila’’ University of Medicine and Pharmacy (Bucharest, Romania). All animals were kept under a rigorous cleaning and hygiene program and were monitored daily. Rats received standard chow and water *ad libitum*. The study was approved by the Ethics Committee from ’’Carol Davila’’ University of Medicine and Pharmacy in accordance with the EU Directive 2010/63 approved in 2014 regarding the protection of animals used for experimental and other scientific purposes. Animals were stratified by blood glucose and body weight values prior to randomization.

#### Treatment with streptozotocin and retroviral transduction with pLPCX-EDEM1

Rats were intraperitoneally injected with freshly prepared streptozotocin (STZ) solution (60g/kg rat) in 0.1 M citrate buffer (pH 4.5).^41^^,^^42^ Blood glucose was measured daily after over-night fasting by tail vein bleed with a glucometer. Rats with a blood glucose concentration over 250 mg/dl at 48h from the STZ injection were used in the experiment. Animals were randomly separated into two groups: first group, N= 9 diabetic rats, was treated with the pLPCX-EDEM1 retrovirus (10^3^ pfu); second group, N=4 diabetic rats, was treated with empty vector (pLPCX). Four non-diabetic control rats were used as negative control. The glucose level was monitored daily for nine days, after which the rats were killed by cardiac puncture under isoflurane anaesthesia.

#### Glucose tolerance test

Wister rats were fasted overnight and glucose level in blood was determined from tail vain blood at time 0. The rats were then injected with 1g/kg (body weight) of glucose. Blood sample were collected after 30, 60, 120 minutes after the glucose administration and glucose level was determined by a glucometer (FreeStyle Optium). The glucose tolerance test was performed before and after treatment with EDEM1.

#### Histopathological analysis

The pancreas was excised after decapitation, washed with saline solution and fixed in 10% formalin for staining tehnique with hematoxylin and eosin (H&E) and histopathological examination. The tissues were processed, embedded in paraffin wax and sectioned with a microtome to obtine 4-5μm thick paraffin sections and then stained with H&E.

#### Data analysis

The data are presented as means ± SEM, were analysed by Student's t test and one-way ANOVA. P≤ 0.05 was considered significant. GraphPad PRISM was used for statistically significant.

## Data Availability

• Original Western blots are publicly available in Mendeley Data https://doi.org/10.17632/txxsv97xvv.2 • Any additional information required to reanalyze the data reported in this paper is available from the lead contact upon request.
