# Supplementary material for: EDEM1 regulates the insulin mRNA level by inhibiting the endoplasmic reticulum stress-induced IRE1/JNK/c-Jun pathway
Source: iScience. 2023 Sep 16;26(10):107956. doi: 10.1016/j.isci.2023.107956 (PMC10562789; doi:10.1016/j.isci.2023.107956)
Supplement: Document S1. Figures S1–S7 and Tables S1–S3 [file mmc1.pdf]

iScience, Volume *26*

Supplemental information

EDEM1 regulates the insulin mRNA level

by inhibiting the endoplasmic reticulum

stress-induced IRE1/JNK/c-Jun pathway

Petruta R. (Flintoaca) Alexandru, Gabriela N. Chiritoiu, Daniela Lixandru, Sabina Zurac, Constantin Ionescu-Targoviste, and Stefana M. Petrescu

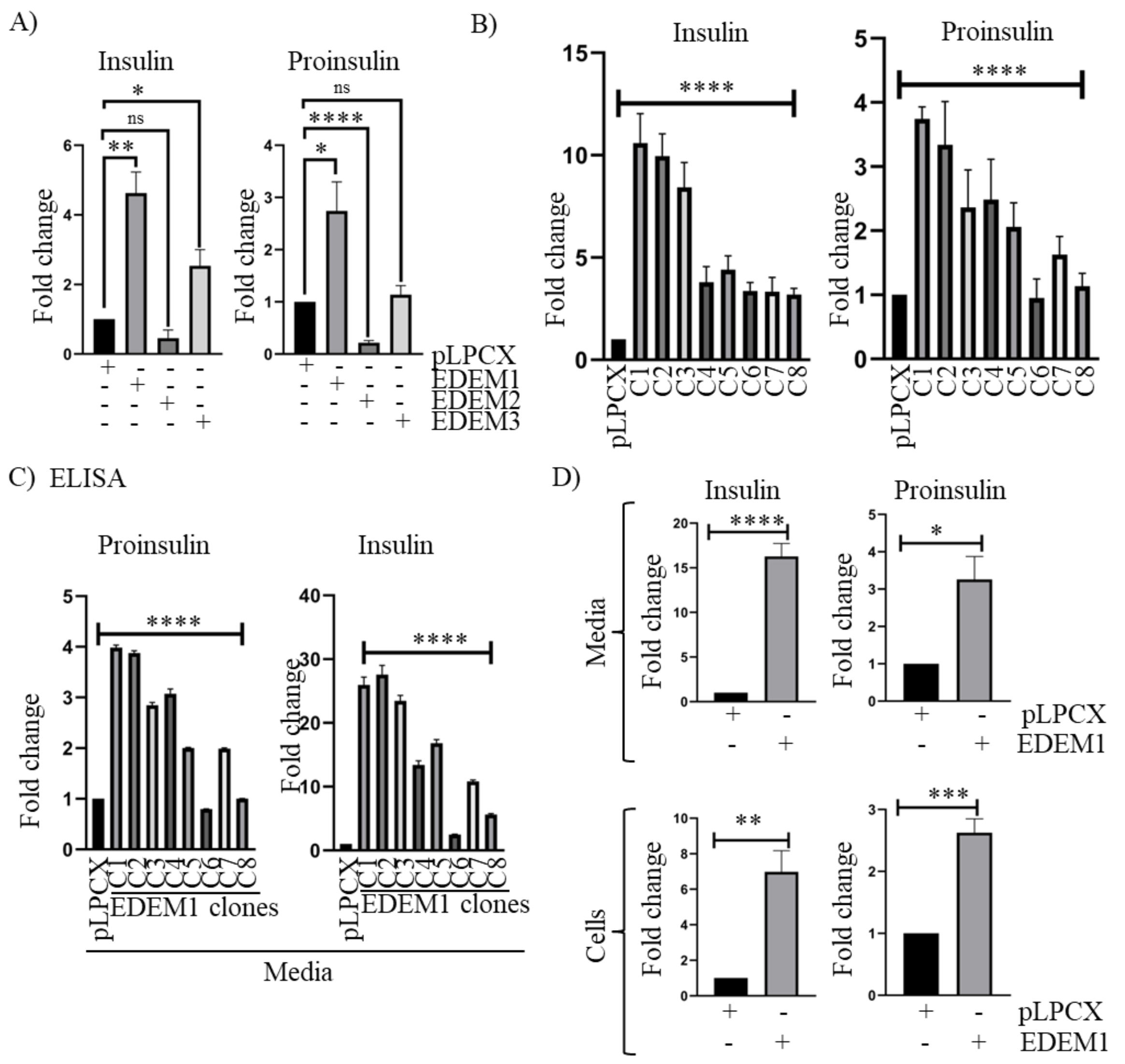

Figure S1. Related to Figure 1. EDEM1 overexpression increases insulin content and secretion in insulinoma cells. A) Proinsulin and insulin bands from Western Blotting like that shown in Figure 1A were quantified using ImageJ at 14mM glucose. B) Proinsulin and insulin bands from Western Blotting like that shown in Figure 1B were quantified using ImageJ. C) INS-1E-Ctrl and eight cell clones showing different levels of EDEM1 protein were grown in RPMI-1640 medium with 11.2 mM glucose for 72h, proinsulin and insulin secretion were quantified by ELISA assay. D) Proinsulin and insulin bands from Western Blotting like that shown in Figure 1C were quantified using ImageJ at 14mM glucose.

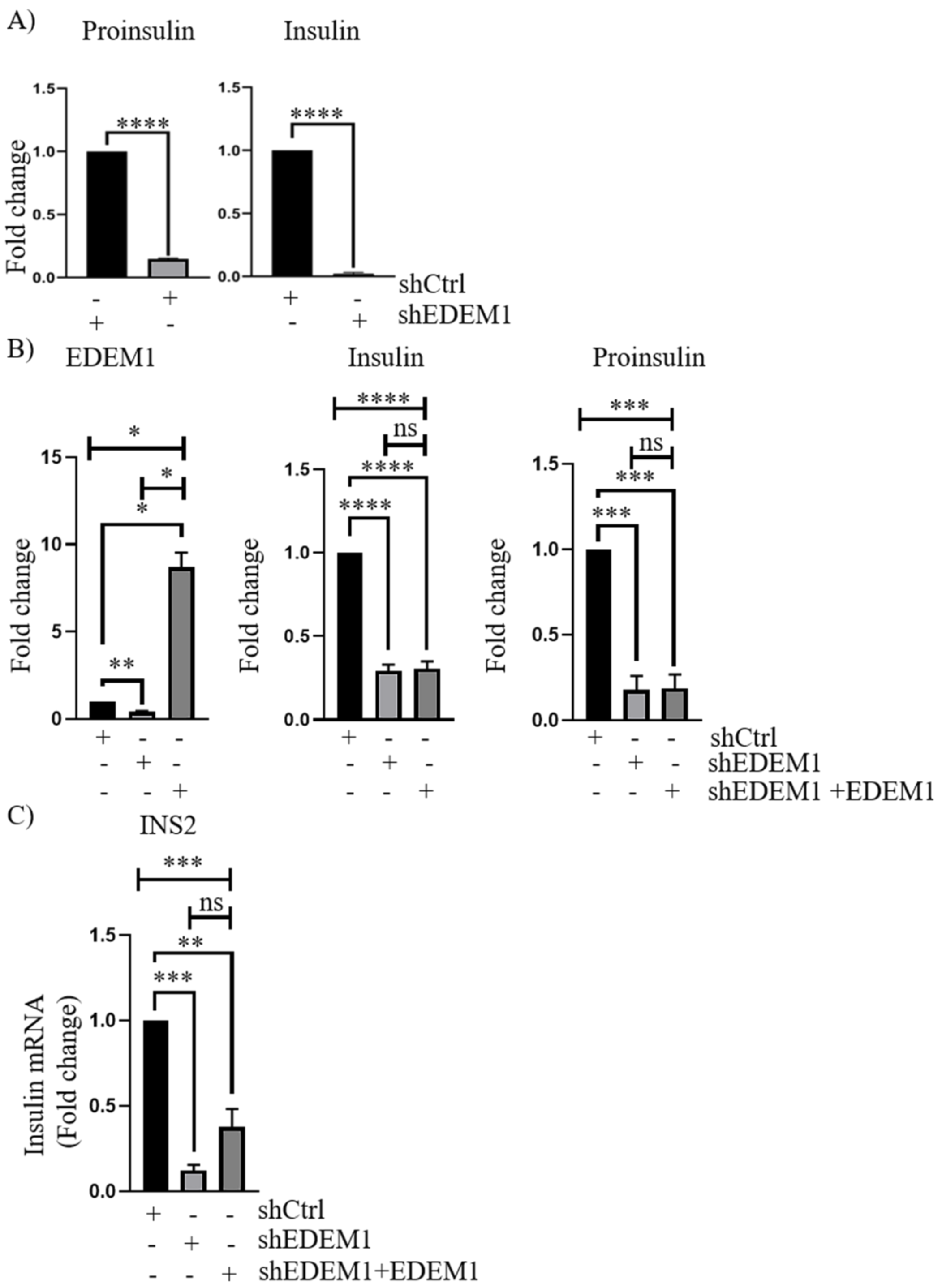

Figure S2. Related to Figure 2. EDEM1 silencing decreases insulin content and secretion in INS-1E cells.A) Proinsulin and insulin bands from Western Blotting like that shown in Figure 2A were quantified using ImageJ at 14mM glucose. B) Quantification of EDEM1, proinsulin and insulin bands from Western Blotting. C) For EDEM1 rescue, shEDEM1 cells were transfected with pLPCXpcx-EDEM1 for 48h and total RNA was isolated and performed Real-Time PCR to determine the insulin mRNA level.

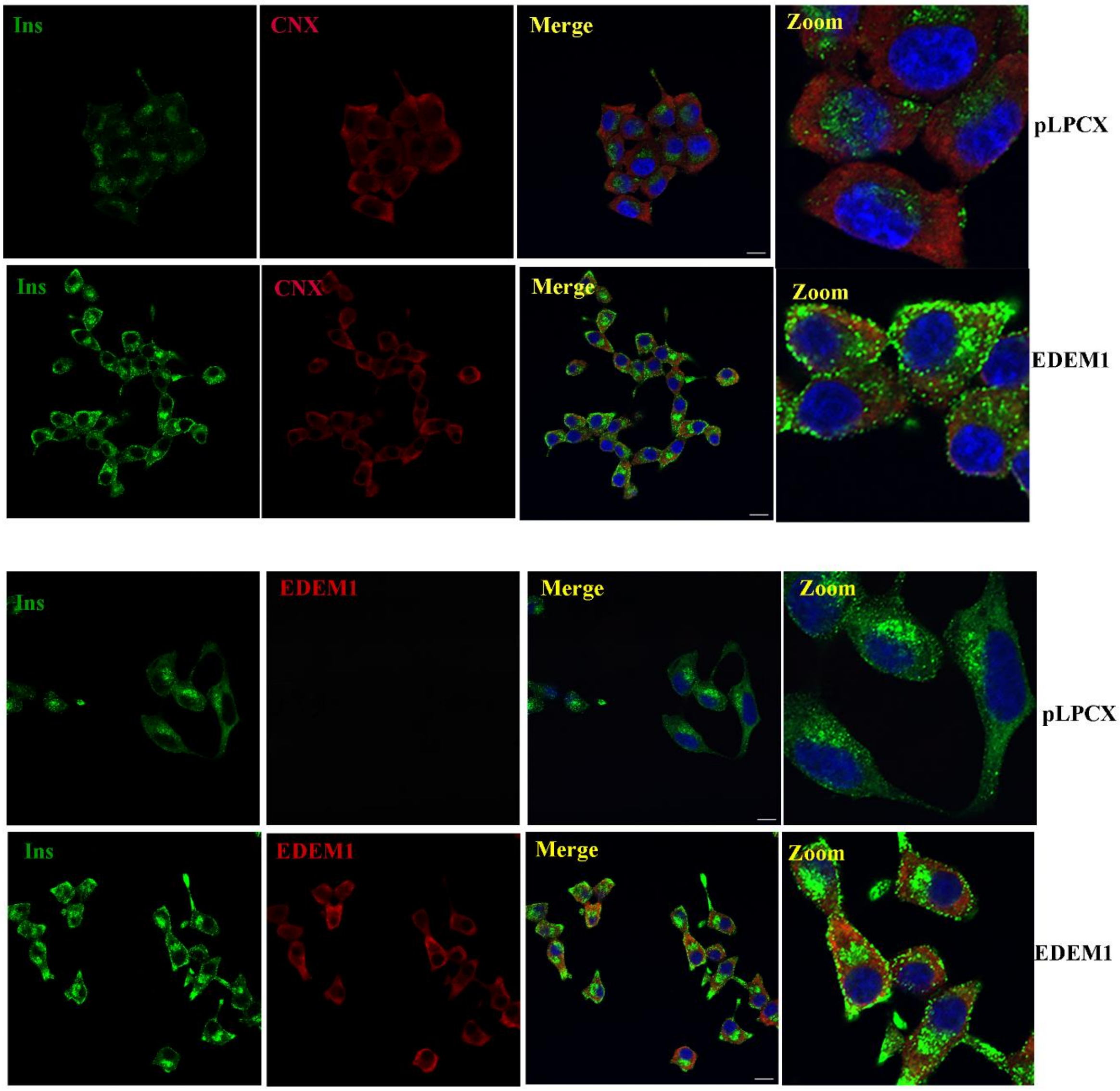

Figure S3. Related to Figure 3. Confocal analysis of INS-1E-pLPCX and INS-1E-EDEM1 cells starved 1h and stimulated with 14mM glucose for 24 h, fixed and stained for insulin (anti-insulin B antibody), EDEM1, calnexin-left panel. Scale bar is 10µm.

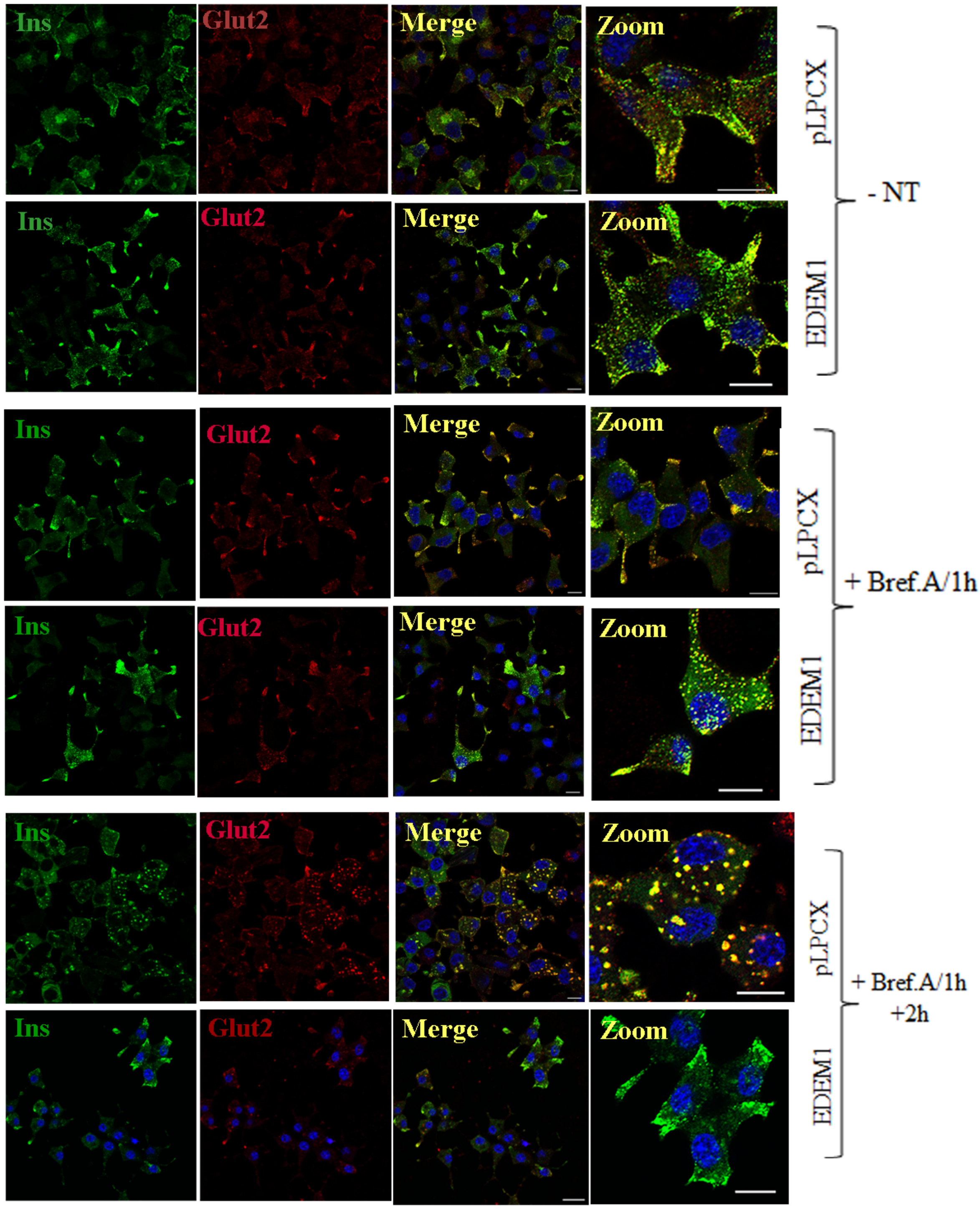

Figure S4. Related to Figure 3. INS-1E-pLPCX and INS-1E-EDEM1 cells were treated or not (NT) with Brefeldin A (Bref.A) for 1h (chase point 0h) and chased for 2h following Brefeldin A removal. Cells were fixed with 4% PFA and stained with Insulin B and Glut2 antibodies. Scale bar is 10µm.

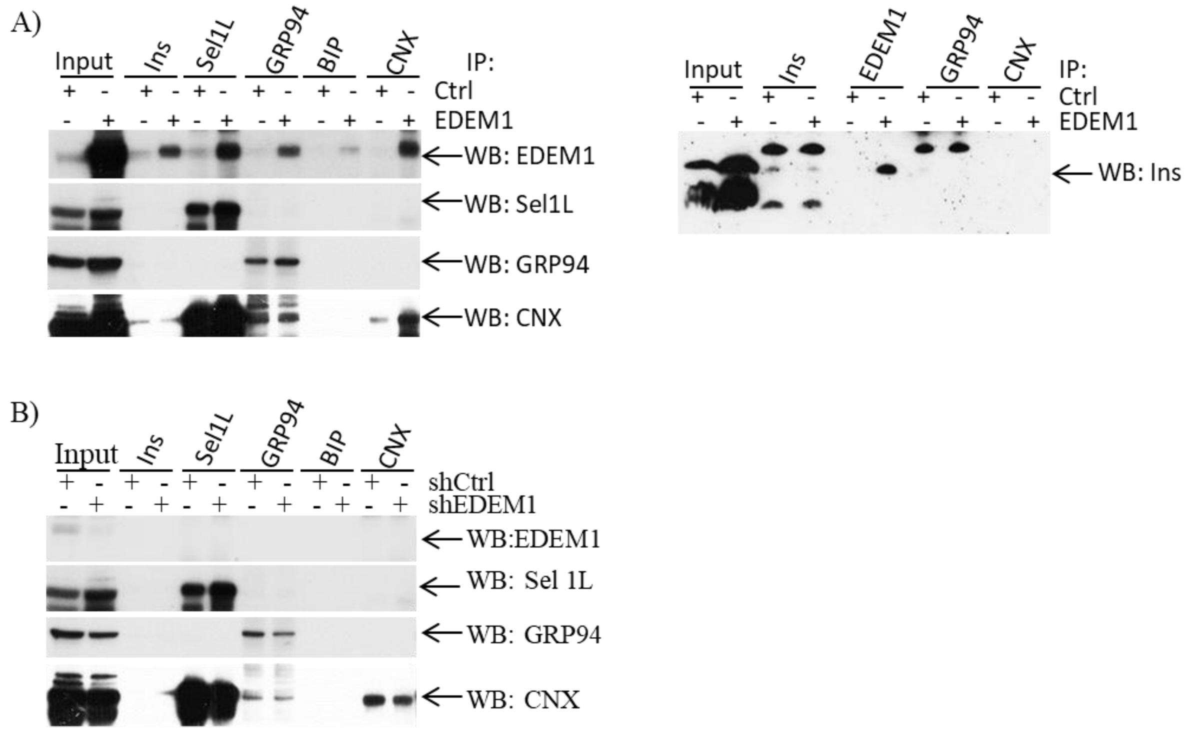

Figure S5. Related to Figure 3. EDEM1 interacts with proinsulin in pancreatic beta cells. INS-1E-Ctrl and INS-1E-EDEM1 cells (A) and INS-1E-shCtrl and INS-1E-shEDEM1 cells B) were grown in medium with 11.2 mM glucose for 72h and cell lysates were incubated overnight with the indicated antibodies. Next day antigen-antibody complex has been related to protein A and/or G-Sepharose and binding proteins were eluted and immunoblotted with indicated antibodies. Data are representative of at least three independent experiments.

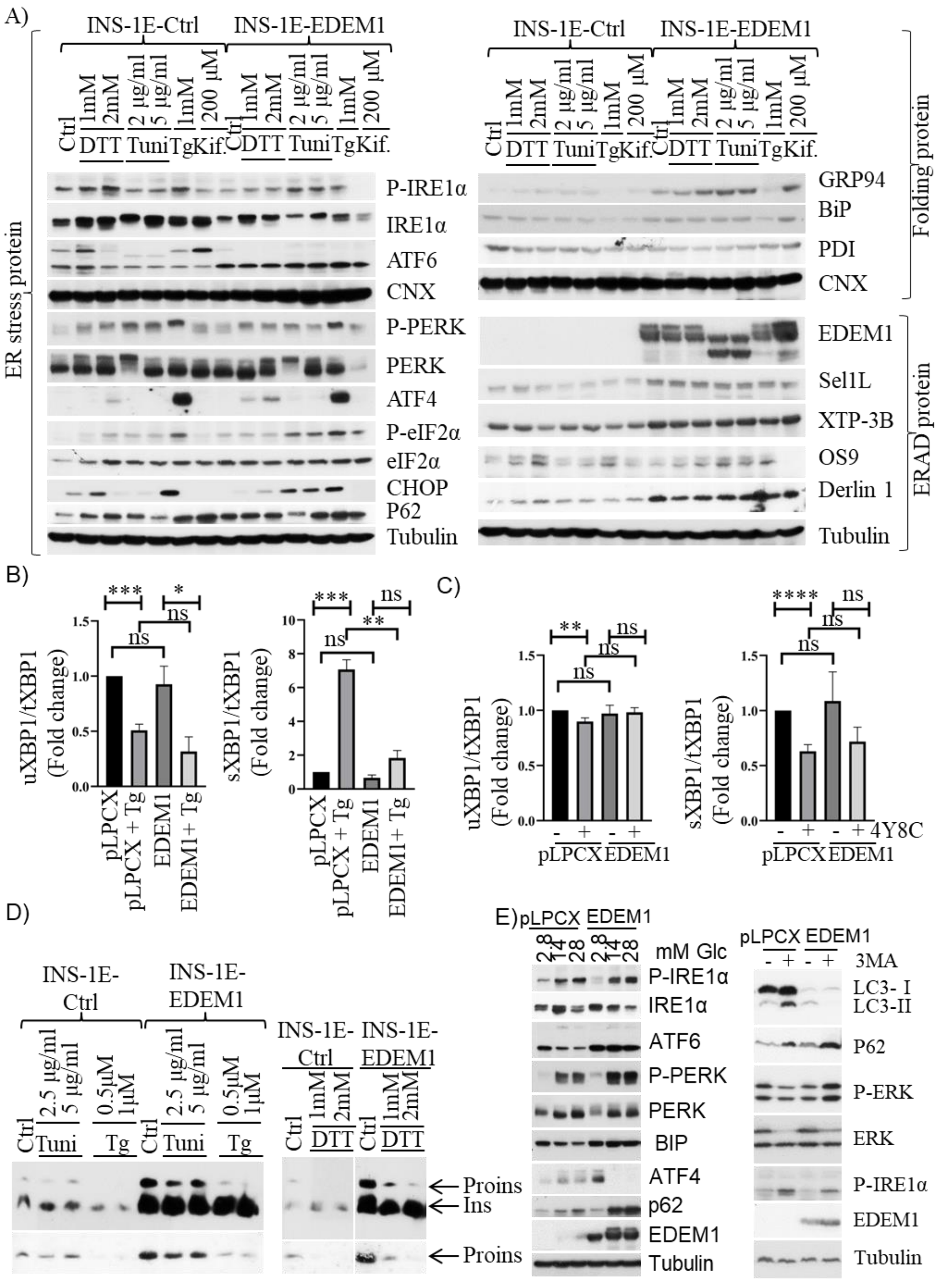

Figure S6. Related to Figure 6. Overexpression of EDEM1 modulate UPR during ER stress. A) Immunoblots from lysates of INS-1E-Ctrl and INS-1E-EDEM1 cells treated with tunicamycin and thapsigargin and DTT for 6h. Equal amounts of protein were resolved by SDS-PAGE and immunoblotted with indicated antibodies. INS-1E-Ctrl and INS-1E-EDEM1 cells were treated or not with thapsigargin (Tg) for 6h and total RNA was isolated and performed Real-time PCR to determine the level of tXBP1, uXBP1 and sXBP1, the ratio usXBP1/tXBP1 and sXBP1/tXBP1 is represented in B). As in B) but cells were treated cu 4y8C for 24h and the ratio usXBP1/tXBP1 and sXBP1/tXBP1 is represented in C). *D)* As in *A)* but immunoblotted with Insulin antibodies. E) For direct comparation of UPR induction and the role of EDEM1, INS-1E-Ctrl and INS-1E-EDEM1 cells were incubated with 2.8 mM, 14 mM and 28mM glucose for 24h and analysed by Western Blotting with indicated antibodies. *G)* INS-1E-Ctrl and INS-1E-EDEM1 were seeded 48h before treatment with 3MA. Conversion of LC3B-I to LC3B-II was determined by Western blotting using anti-LC3 antibodies, in the presence or absence of 3MA for 12h, autophagy inhibitors (right panel). Tubulin expression was used as total protein-gel loading control.

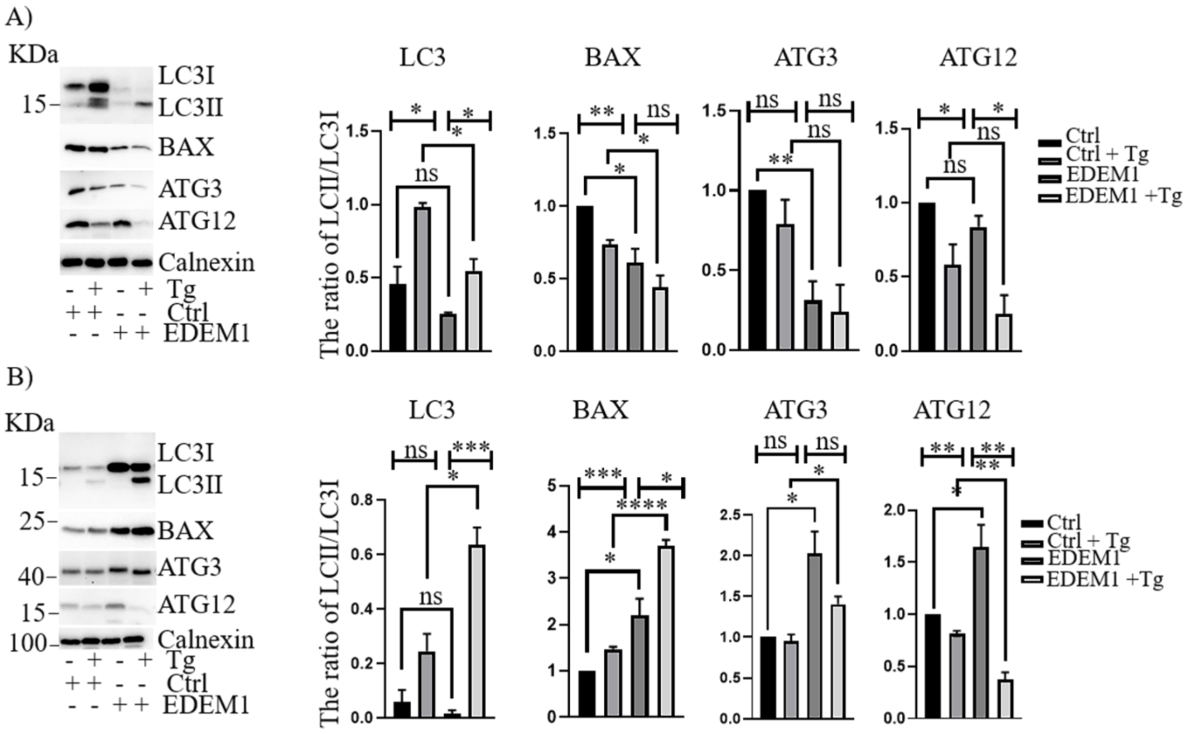

Figure S7. Related to Figure 6 and Figure 7. Overexpression of EDEM1 suppress autophagy process and silencing of EDEM1 activates autophagy during ER stress. A) INS-1E-Ctrl and INS-1E-EDEM1 cells and B) INS-1E-shCtrl and INS-shEDEM1 cells were treated with thapsigargin for 6h. Equal amounts of protein were resolved by SDS-PAGE and immunoblotted with indicated antibodies. Protein bands from Western Blotting were quantified using ImageJ program-right side. Data are representative for three independent experiments. Results are expressed as means ± SEM.

Table S1. Related to Figure 3. shRNA sequences used for EDEM1 silencing in INS-1E cells

Catalog number	shRNA sequences
KR47594 XM_23836 6	TCTCggacaaacgtcttcgggaattCTTCCTGTCAaattcccgaagacgtttgtccCT
KR47594 XM_23836 6	TCTCGtcaacgatgtactaggaaattCTTCCTGTCAaatttcctagtacatcgttgaCT
KR47594 XM_23836 6	TCTCGttgactctctgcaggctttctCTTCCTGTCAagaaagcctgcagagagtcaaCT
KR47594 XM_23836 6	TCTCggagcaacgatacaggattatCTTCCTGTCAataatcctgtatcgttgctccCT

Table S2. Related to Figure 3. Oligonucleotide primer sets for quantitative real-time PCR (RT-PCR)

Gene	5'Forward 3'	5'Reverese 3'
Rat Insulin II	5'-ATCCTCTGGGAGCCCCGC-3'	5'-AGAGAGCTTCCACCAAG-3'
Rat Actin	5'-GCAAATGCTTCTAGGCGGAC-3'	5'-AAGAAAGGGTGTAAAACGCAGC-3'
Total XBP1	5'TGGCCGGGTCTGCTGAGTCCG-3'	5'-ATCCATGGGAAGATGTTCTGG-3'
usXBP1	5'-CAGCACTCAGACTACGTGCG-3'	5'-ATCCATGGGAAGATGTTCTGG-3'
sXBP1	5'-CTGAGTCCGAATCAGGTGCAG-3'	5'-ATCCATGGGAAGATGTTCTGG-3'

Table S3. Related to Figure 5. Number of islets of Langherhans and weight body

Wistar rats	Ctrl	pLPCX	EDEM1	STZ + pLPCX	STZ + EDEM1

Number of islets of Langerhans (average)	19.5	28	25.5	10.25	13.83
Weight body (average)	264	258	270	203	215

Average pancreatic islet numbers of the pancreata analysed in figure 4 (N=3 for all groups).
